# H^+^ exchange and pH profiling of select non-glycolytic cytosolic phosphate transfer and oxidation–reduction reactions

**DOI:** 10.3389/fmolb.2026.1816622

**Published:** 2026-09-21

**Authors:** Robert Robergs, Marek Nalos, Petr Kelbich, Adela Jizerová, Ivana Mervartová, Michal Vostrý, Milan Štengl, Vaclav Babuška

**Affiliations:** 1 Faculty of Health Studies, Jan Evangelista Purkyné University, Ústínad Labem, Czech Republic; 2 Department of Physiology, Faculty of Medicine, Charles University, Pilsen, Czech Republic; 3 Department of Intensive Care Medicine, Pilsen Hospital and Charles University, Pilsen, Czech Republic; 4 Department of Biomedicine and Laboratory Diagnostics, Faculty of Health Studies, Jan Evangelista Purkyné University and Masaryk Hospital in Ústínad Labem, Ústínad Labem, Czech Republic; 5 Department of Environmental Chemistry, Jan Evangelista Purkyné University, Ústínad Labem, Czech Republic; 6 Department of Medical Chemistry and Biochemistry, Faculty of Medicine, Charles University, Pilsen, Czech Republic

**Keywords:** acidosis, alkalosis, bioenergetics, buffering, enzymology, metabolism

## Abstract

**Background:**

Despite animal research of cellular and systemic acidosis for more than 100 years, there is no dominant evidence-based understanding of the cause of metabolic acidosis. Embedded in this crisis state is the understanding that the hydrogen ion (H^+^) involved in some chemical reactions, either as a substrate or product (H^+^ exchange (H^+^_e_)), does not change the pH of biological solutions. This research documents the direct roles of the H^+^_e_ of a selection of non-glycolytic cytosolic chemical reactions in changing the pH of an unbuffered aqueous solution.

**Methods:**

Reagents were prepared using deionized water, substrates at known concentrations, enzymes and needed activator chemicals. The chemical reactions studied were creatine kinase (CKase) (to ATP and CrP), ATPase, hexokinase (HKase), and glucose-6-phosphate dehydrogenase (G_6_PDHase). Each reaction was started by addition of the enzyme, and reagent pH was measured intermittently using a pH electrode. Samples of reagent (200 μL) were acquired for product measurement by spectrophotometry.

**Results:**

Results are presented as start pH:end pH; [Product], mmol·L^-1^; pH:[Product] slope, pH.mmol^-1^.L^-1^: CKase to ATP (7.49:9.51; [1.95]; 1.289); CKase to CrP (8.29:7.89; [0.64]; -1.35); ATPase (8.94:8.23; [0.84]; -0.9385); HKase (8.59:6.55; [2.36];-1.596), G_6_PDHase (7.53:6.89; [1.46]; -0.8006). All chemical reactions that produced carboxylic acids did so as anions, and all resulted in pH responses consistent with the role of the H^+^ as a substrate or product. The sequencing or summing of the CKase, ATPase, HKase and G6PDHase reactions revealed potential for summative computational modelling.

**Conclusions:**

The evidence opposes traditional theories as the sole cause of cellular or systemic acidosis.

## Introduction

The importance of hydrogen ion (H^+^) involvement (H^+^ exchange; H^+^_e_) in the chemical reactions of the animal (heterotrophic: energy released from chemical reactions) cellular metabolism has gained increased attention across the last 3 decades. Previous research has been based on dissociation constants and computational chemistry (Kushmerick, 1997; Vinnakota et al., 2006; Robergs, 2017), and from this evidence it has been proposed that such chemical reaction H^+^_e_ should mean that these reactions have potential to directly alter the pH of their aqueous solution (Robergs, 2017; Robergs, 2019; Robergs, 2021; Torrens et al., 2023). For example, some reactions could increase pH (where H^+^ are consumed in the reaction), other reactions could lower pH (where H^+^ are released in the reaction), while others (no H^+^ involvement as a substrate or product in the reactions) should have no influence on pH other than from minor adjustments caused by the differences in the dissociation constants between substrates to products (Robergs, 2017). Such evidence and interpretations oppose the traditional acid–base chemistry and physiology construct of the cellular production of acid molecules (Hill and Lupton, 1923; Kreisberg, 1980; Katz and Sahlin, 1988; Sirker et al., 2012; Seheult et al., 2017), in addition to alternative physicochemical theories of the cause of cellular and systemic acidosis (Stewart, 1983; Sirker et al., 2012). However, the former acid interpretation requires clarification. Nomenclature within acid-base chemistry is problematic as there are definitions of an acid molecule that can be contradictory in biological applications. In a structural definition, where an acid molecule has an acid functional group, the carboxylic acids of the cellular metabolism are a prime example. Moreover, an acid is a molecule that, when dissolved in an aqueous solution at pH 7, causes the release of a H^+^ in a pH-dependent (dissociation constant) manner, causing a decrease in pH. Historically, this latter definition was definitive (Lesney, 2003; Kragh and Sorenson, 2025). Nevertheless, the study of chemical reactions in aqueous solutions *in vitro*, or within biological systems, is known to produce acid metabolites by structure (e.g., carboxylic acids) but in anion form (carboxylates); as such, they are incapable of releasing a H^+^ and lower pH.

Before elaborating on the purposes and methods of this research, it is very important to document some historical and recent research on H^+^ involvement in chemical reactions, and specifically to contrast animal cellular biochemistry with plant cellular (autotrophic: energy obtained from sunlight via photosynthesis) biochemistry (Lehninger, 1960; 1961; Mitchell, 1976). While animal cellular acid–base chemistry has been constrained for more than 150 years by the assumption that cells produce metabolic acids and that these acids induce acidosis based on pH-dependent H^+^ dissociation, the progress within plant biochemistry differs. As plants use sunlight and not chemical reactions as their predominant energy source, it seems that the disciplines linked to plant-based bioenergetics have been more free to investigate alternate biochemical mechanisms that release H^+^. For example, as early as the 1970s, plant biochemical and molecular biology research has provided evidence of the direct impact of chemical reactions on altered chemical reaction H^+^_e_, independent of acid production (Sakano, 1998). Such early research of plant-based pH regulation focused on the role of H^+^-generated electrochemical gradients, which, in turn, provide the free energy for many biochemical cell work systems. However, even early theories of the cause of the acidosis within plant biochemistry were contradictory: malate production, originally believed to cause a decrease in pH, akin to muscle lactic acid production, actually consumes a H^+^ (similar to the LDH reaction to lactate production) (Sakano, 1998). The solution to this dilemma was based on the interpretation of how the phosphate-transfer and oxidation–reduction chemical reactions of glycolysis that produce carboxylic acids do so with these acids being in their anionic, not acidic, structure. This means that the reactions that result in the covalent release of a H^+^ (e.g., hexokinase, PFK, and G_3_PDH reactions) do so based on the role of a H^+^ as a product (Sakano, 1998). Such evidence revealed the previously stated inconsistency in the terminology of the biological applications of acid–base chemistry.

It is interesting that the plant-based cellular biochemistry of pH regulation recognized the direct importance of chemical reaction H^+^_e_, whereas animal cellular biochemistry remained blind to this evidence. Further evidence of such alternative plant-based biochemistry which depends on the chemical reaction H^+^_e_ is the pH regulation of the thylakoid compartment of chloroplasts (Trinh and Masuda, 2022). Such a system links H^+^ channels to H^+^ gradients and free-energy-generating charge potentials, devoid of any acid metabolite production. In this context, the increasing _a_H_3_O^+^ and potential gradients within and between compartments within animal cells may be part of a similar system of free energy generation, not that dissimilar to the Mitchell oxidative phosphorylation system linking mitochondrial oxidation to the H^+^ gradient between the inter-membranous space and matrix, further coupled to the free energy need of ATP formation and reduction of molecular oxygen to water. Indeed, as early as 1976, Mitchell referred to the free energy attainable from gradients in _a_H_3_O^+^ as “proticity” and hinted that such systems may not be limited to within mitochondria. More recently, Roosterman and Cottrell (2025) proposed, based on fundamental principles of non-steady state thermodynamics, that the increased _a_H_3_O^+^ activity resulting from increased energy demand in animal cells (e.g., skeletal muscle fibers) may couple different reactions where a H^+^ is a product in one and a substrate in another. It is highly likely that animal-based biochemistry has misunderstood the H^+^ involvement within acid-base chemistry and the biochemistry of cellular energy transfer.

Recently, results from the measurement of pH profiles of the lactate dehydrogenase (LDHase) reaction (Robergs et al., 2026) have provided experimental cause–effect evidence for the validity of the direct influence of the H^+^_e_ of the LDHase reaction to changes in the pH of the aqueous solution. For example, the coupled oxidation of NADH to NAD^+^ and reduction of pyruvate to lactate, which is the intrinsically exergonic direction of the reaction (Table 1), consumed a H^+^ from solution for each lactate produced and caused an increase in the pH of the aqueous reagent. Conversely, the coupled oxidation of lactate to pyruvate and reduction of NAD^+^ to NADH released a H^+^ for every pyruvate produced and caused a decrease in the pH of the aqueous reagent.

**TABLE 1:** Details of the five cytosolic chemical reactions of this research.

Chemical reaction	^*^ΔG°’	^*^ΔG	∼H^+^_e_^#^	
			pH = 6.0	pH = 7.0
$CrP+ADP+H^{+}\overset{CKase}{\to }Cr+ATP$	−12.6^ϕ^	−0.1^ϕ^	0.8023	0.9690
$Cr+ATP\overset{CKase}{\to }CrP+ADP+H^{+}$	12.6^ϕ^	0.1^ϕ^	−1.17	−1.03
$ATP+H_{2}O\overset{ATPase}{\to }ADP+HPi+H^{+}$	−32.42^λ^	−37.41^λ^	−0.0097	−0.6635
$Glucose+ATP\overset{HKase}{\to }G_{6}P+ADP+H^{+}$	−16.7^ϕ^	−30.98^ε^	−1.258	−1.850
G6P+NADP+→G6PDHaseP6Gl+NADPH+H+	−7.51^λ^	−46.2^λ^	−1.47^δ^	−1.10^δ^

ΔG°’ = standard Gibb’s free energy exchange; ΔG°’ = absolute (*in vivo*) Gibb’s free energy exchange; HPi = HPO4^-2^; * = kJ M^-1^ and expressed in the presented left to right direction of the chemical reaction; ∼ = fractional (< or >1) where +’ve = H+.

consumption and -‘ve = H+ release; #Robergs (2017), other than for the HKase, reaction which was recalculated due to a detected error (omission of the H+ release) in the published data for this reaction; ϕ from Nelson and Cox (2021) and Goldberg (1975); λ from Li et al. (2011); ε calculated based on [glucose] = 0.15 mM, [ATP] = 8.5 mM, [G6P] = 0.1 mM, [ADP] = 0.05 mM; φ calculated as per Robergs (2017); δ calculated as per Robergs (2017) with the pK of 6PGl, 4.995 from Li et al. (2016).

The LDH reaction is oxidation-reducing, and as previously explained, other oxidation–reduction reactions occur in the cytosol of animal cells, such as in glycolysis (glyceraldehyde-3-phosphate dehydrogenase reaction) and in the redirection of glucose-6-phosphate (G_6_P) from glycolysis to the pentose phosphate pathway (G_6_P dehydrogenase (G_6_PDHase) reaction) (Voet and Voet, 2018; Nelson and Cox, 2021). Prior computational chemistry research has also shown that some of the phosphate transfer reactions release or consume a H^+^ in their exergonic direction (Table 1; Figure 1) (Kushmerick, 1997; Vinnakota et al., 2006; Robergs, 2017; 2019; 2021).

**FIGURE 1:**
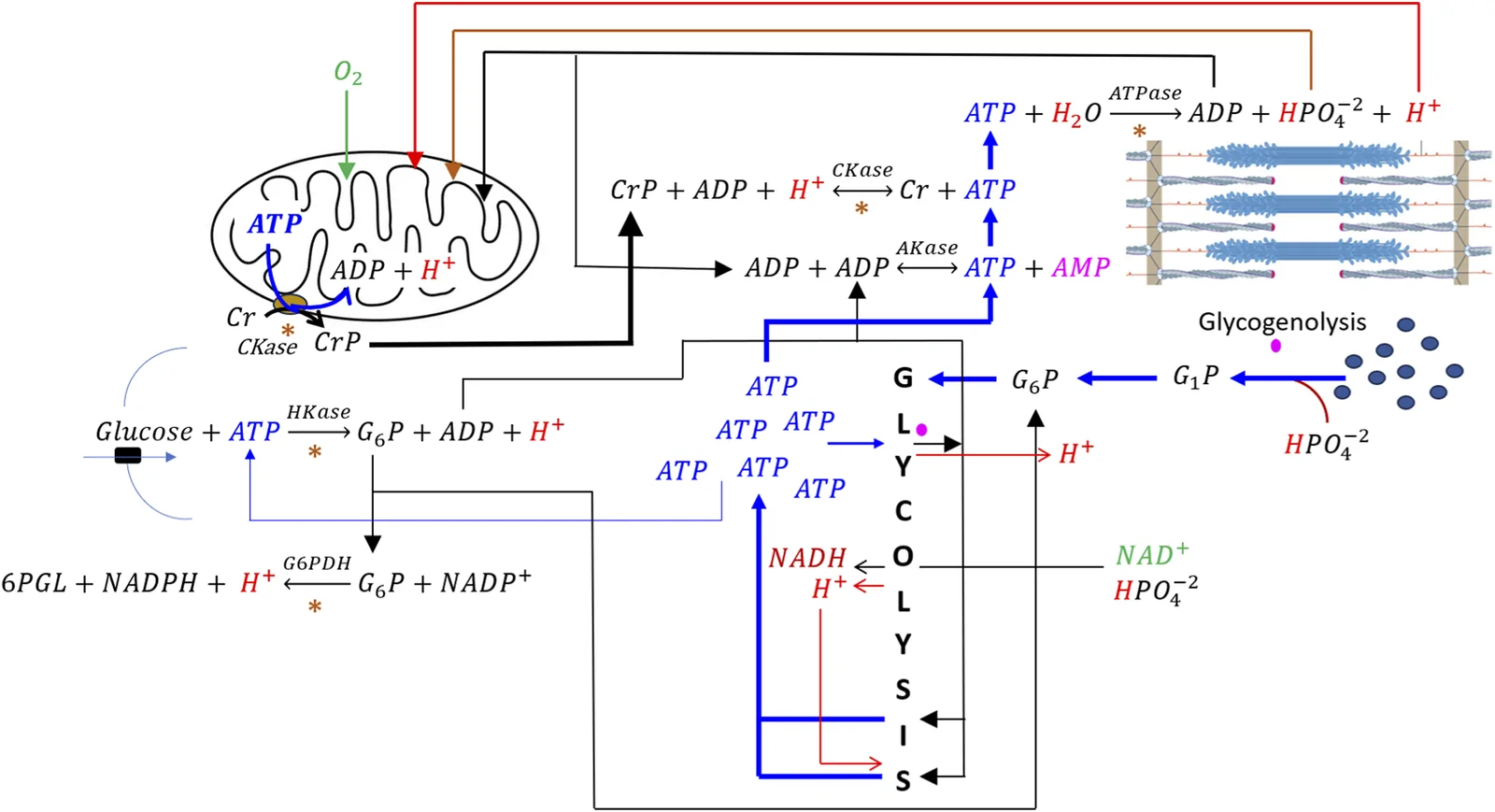
Simplified diagram focusing on chemical reactions of heterotrophic cells, such as skeletal muscle fibers, involved in the free energy transfer to and from ATP in the reactions of the phosphagen system, which, in turn, is linked to free energy transfer to and from glycolysis and mitochondrial respiration. Orange asterisk (*) indicates chemical reactions involved in this research. Magenta filled circles (•) indicate allosteric enzyme regulation by AMP.

It is logical to expand on the experimental measurement of pH changes during chemical reaction H^+^_e_ for other oxidation–reduction reactions to that of LDHase, along with some of the phosphate transfer reactions of cellular energy catabolism. The main non-glycolytic oxidation–reduction and phosphate-transfer reactions pertinent to cell free energy transfer in animal cells are presented in Table 1 and Figure 1. Our intent is to focus on non-glycolytic chemical reactions, as our future research will explore specific reactions of glycolysis in the context of the entire glycolytic pathway.

### Research problem

Traditional accounts of pH changes during cellular energy catabolism in animals have been dependent on the assumption that cells produce metabolic acids, and it is these reactions that collectively account for the cellular and systemic acidosis that can develop during conditions of high cellular energy demand and/or deficient oxygen provision (e.g., caused by severely lowered tissue or organ perfusion or mitochondrial dysfunction) (Hill and Lupton, 1923; Katz and Sahlin, 1988; Lesney, 2003; Sirker et al., 2012; Aiken, 2013; Seheult et al., 2017). Inherent to this interpretation is the assumption that chemical reactions that have a H^+^ as a substrate or product do not alter the pH of biological solutions. Such an assumption also forms the core feature of Stewart’s physical chemical theory for acidosis in animal biological systems (Stewart, 1983). Recent experimental research has documented that this is not the case for the LDHase reaction, where pyruvate reduction to lactate increased pH, while lactate oxidation to pyruvate decreased pH (Robergs et al., 2026). Such results are further supported by more than 50 years of plant biochemistry research into cellular energy transfer (Sakano, 1998; Mitchell, 1976) and indicate the need for research that profiles the pH changes that occur in an aqueous reagent for other chemical reactions that involve H^+^_e_.

### Purpose statement

We aimed to study the *in vitro*-pH-to-time profiles of a selection of chemical reactions, external to glycolysis (Table 1; Figure 1) that support cellular free energy transfer within animal biological systems, such as the creatine kinase (CKase) (in both directions) and ATPase reactions. Furthermore, as the hexokinase (HKase) reaction is an important phosphate transfer reaction to support glycolysis and the glucose-6-phosphate dehydrogenase (G_6_PDHase) reaction re-directs glucose-6-phosphate (G_6_P) to the pentose-phosphate pathway, both reactions are also included in this research. The inclusion of these chemical reactions in this research is further reinforced by the G_6_PDHase reaction, like the LDHase reaction, being an oxidation–reduction reaction involving H^+^_e_. In addition, the HKase and G_6_PDHase reactions are both involved in the enzymatic spectrophotometric assay of glucose, ATP, CrP, and G_6_P. As all such assays were also a part of this research for the measurement of the concentration of pertinent products ([Product]), it was logical to also investigate their involvement in the H^+^_e_ of chemical reactions.

Consequently, the purpose of this research is to 1) measure the pH and [H^+^] (more correctly referred to as the “hydronium ion concentration” [H_3_O^+^] or “activity” _a_H_3_O^+^) to time profiles of an unbuffered *in vitro* reagent for the five chemical reactions of interest (Table 2) at different substrate concentrations ([S]), initial pH (pH_start_), and enzyme activities (Table 3); 2) quantify [Product] from a representative data set of each reaction; 3) measure the pH and [H^+^] to [Product] profiles from a representative dataset of these reactions; and 4) ascertain the quantum nature of the pH changes through different combinations of the chemical reactions when sequenced, as well as when operating simultaneously. Such research will provide the evidence for how these chemical reactions can or cannot directly alter the pH of their aqueous environment. In addition, the methodology will establish the quantum nature or its absence of the pH changes in more complex reaction conditions involving the production of a H^+^ buffer (ATPase reaction producing inorganic phosphate (HPO_4_^−2^)) and where multiple reactions occur in sequence or simultaneously. As the substrates to products of chemical reactions are invariant (they are the same in an *in vitro* reagent as they are in a cell) and where the reactions studied involve a H^+^ as either a substrate or product, the results inform the potential roles of these reactions to alter cellular acid-base balance during conditions of altered cellular energy demand.

**TABLE 2:** Details of the biochemicals used in the chemical reaction pH-to-time profiles and product assays.

Chemical	Supplier	Code	Mol. Weight*
*Biochemical*			
Na^+^-creatine phosphate	Merck	27920	327.14
H_2_O-creatine	Fisher	B25009-22	131.13
Mg^+2^-ATP	Merck	A9187	507.18
Mg^+2^-ADP	Merck	A2754	427.20
MgCl_2_	Merck	8.14733	95.21
NaOH (50% solution in H_2_O)	Merck	417661	56.11
KOH (45% by wt. solution in H_2_O)	Merck	415413	40.0
Hydrochloric acid (37% in H_2_O)	Merck	258148	36.46
Tris-base^^^	Merck	93352	121.14
Tris-HCl^^^	Merck	T3253	157.60
Dithiothreitol^^^	Merck	D0632	154.25
Na^+^-NADP^+^^^^	Merck	481971-M	765.39
D-glucose^^^	Merck	158968	180.16
*Enzymes*			
Creatine kinase, lyophilized powder, 1,000 U (from rabbit muscle; EC: 2.7.3.2)	Merck	C3755	
Na^+^,K^+^-ATPase, 5 U, (from porcine cerebellum; EC: 3.6.1.3)	Merck	A7510	
Hexokinase, 1,000 U (from yeast type F-300; EC: 2.7.1.1)	Merck	H4502	
Glucose-6-phosphate dehydrogenase, 500 U (from *Leuconostoc mesenteroides*; EC: 1.1.1.49)^	Merck	346774	

* grams·M-1; ^ used for the spectrophotometric assay of [Product] (G_6_P or ATP).

**TABLE 3:** Details of the reagent compositions of the five chemical reactions of this research, in addition to added methodological features such as the substrate concentrations ([S]), enzyme activities, and the pH values of the reagent for initial conditions (pH_i_), after adjustment (pH_start_), and after enzyme addition and 20 min of the reaction (pH_end_). The peak product accumulation ([P] mmol.L^-1^) for the subset of reactions that involved such assays is also provided.

Direction→Code [P] = mM⋅L^-1^	Substrate*	Amount (g; mL^^^)	[S] (mM⋅L^-1^)^	Enzyme (U)	Vol_start_ (mL) ^^^	pH_i_	pH_start_^#^	pH_end_
*CKase reaction*								
CKATP1	CrP	0.1316	14.27	65	43.72	4.56	5.92	6.63
	ADP	0.1731	8.78					
	KOH^∼^	3.72 mL						
CKATP2	CrP	0.0680	5.05	30	41.2	4.79	6.55	7.06
	ADP	0.0847	4.81					
	KOH^∼^	1.2 mL						
CKATP3	CrP	0.0407	4.79	30	26.0	4.9	6.48	7.11
	ADP	0.0513	4.62					
	KOH^∼^	1.0 mL						
CKATP4	CrP	0.0838	9.56	50	26.8	4.88	6.40	7.06
	ADP	0.1054	9.21					
	KOH^∼^	1.8 mL						
CKATP5	CrP	0.0424	4.97	100	26.1	4.90	6.47	7.67
	ADP	0.0552	4.95					
	KOH^∼^	1.1 mL						
CKATP6	CrP	0.0826	9.99	60	25.03	5.10	8.21	9.46
	ADP	0.0542	4.99					
	NaOH^∼^	1.0 mL						
	KOH^∼^	1.03 mL						
CKATP7	CrP	0.0243	3.10	70	24.0	4.70	7.49	9.51
[P] = 1.95	ADP	0.0327	3.19					
	NaOH^∼^	1.0 mL						
CKCrP1	Cr	0.0307	4.92	50	41.8	3.97	8.17	7.09
	ATP	0.1029	4.85					
	KOH^∼^	1.8 mL	1.80 mL					
CKCrP2	Cr	0.0197	4.91	15	26.98	4.10	8.03	7.78
	ATP	0.0651	4.76					
	KOH^∼^	1.98 mL						
CKCrP3	Cr	0.0188	4.58	100	27.5	4.16	8.13	7.83
	ATP	0.0657	4.71					
	KOH^∼^	2.5 mL						
CKCrP4	Cr	0.0378	10.09	50	25.12	4.02	8.3	7.47
	ATP	0.0643	5.05					
	KOH^∼^	1 mL						
	NaOH^∼^	1 mL						
	HCl`	0.1 mL						
CKCrP5	Cr	0.02014	4.65	60	29.0	3.93	9.23	8.34
	ATP	0.1355	9.21					
	NaOH^∼^	2.0^^^ mL						
	KOH^∼^	1.0^^^ mL						
CKCrP6	Cr	0.0198	5.20	50	25.55	4.0	8.07	7.88
	ATP	0.0638	4.93					
	NaOH^∼^	0.5 mL						
	KOH^∼^	1.8 mL						
CKCrP7	Cr	0.0337	8.72	90	25.95	4.03	8.29	7.89
[P] = 0.64	ATP	0.0655	4.99					
	MgCl_2_^∼^	1.25 mL						
	NaOH^∼^	2.0 mL						
	KOH^∼^	1.7 mL						
*ATPase Reaction* ^ø^								
ATP1	ATP	0.2183	10.34	5	41.63	3.90	9.52	9.43
	NaOH^∼^	0.0 mL						
	KOH^∼^	0.0 mL						
ATP2	ATP	0.1009	4.82	5	41.27	3.92	8.40	6.92
	NaOH^∼^	1.0 mL						
	KOH^∼^	1.0 mL						
ATP3	ATP	0.0648	5.01	2	25.5	4.14	8.77	8.73
	NaOH^∼^	0.0 mL						
	KOH^∼^	2.5 mL						
ATP4	ATP	0.0638	4.93	3	25.5	3.95	8.83	7.57
	NaOH^∼^	1.0 mL						
	KOH^∼^							
ATP5	ATP	0.0647	4.81	2.5	26.5	3.98	7.25	7.28
	MgCl_2_^∼^	1.25 mL						
	NaOH^∼^	1.5 mL						
ATP6	ATP	0.0641	5.13	2.5	25.05	4.00	7.19	7.34
	MgCl_2_^∼^	1.25 mL						
	NaOH^∼^	1.0 mL						
	KOH^∼^	0.8 mL						
ATP7	ATP	0.0634	4.60	2.5	27.15	3.99	8.94	8.23
[P] = 0.84	MgCl_2_^∼^	1.25 mL						
	NaOH^∼^	3.0 mL						
	KOH^∼^	1.8						
*HKase reaction*								
HK1	Glucose	0.0727	9.95	10	42.55	3.86	9.36	6.45^**^
	ATP	0.2029	9.87					
	KOH^∼^		2.7 mL					
	HCl∼		0.8 mL					
HK2	Glucose	0.0405	5.46	20	41.20	3.95	7.80	5.95
	ATP	0.1029	4.92					
	MgCl_2_	2.0 mL						
	KOH^∼^	2.0 mL						
	HCl∼	0.2 mL						
HK3	Glucose	0.0139	3.21	15	24.0	4.00	8.59	6.55
[P] = 2.36	ATP	0.0380	3.12					
	MgCl_2_	1.25 mL						
	NaOH^∼^	1 mL						
HK4	Glucose	0.0142	3.14	15	25.1	3.91	8.50	7.04
	ATP	0.0385	3.02					
	MgCl_2_	1.5 mL						
	NaOH^∼^	0.8 mL						
	KOH^∼^	1.3 mL						
HK5	Glucose	0.0144	3.18	15	25.22	4.01	8.47	7.03
	ATP	0.0386	3.02					
	MgCl_2_	1.5 mL						
	NaOH^∼^	1.0 mL						
	KOH^∼^	1.22 mL						
*G*_*6*_ *PDH reaction*								
G6P1	G_6_P	0.0402	4.75	25	27.75^°^	3.25	8.11	6.75
	NADP^+^	0.0850	3.70					
	NaOH^∼^	1.5						
	KOH^∼^	0.75						
G6P2	G_6_P	0.0408	4.45	20	28.5	3.25	7.53	6.89
[P] = 1.46	NADP^+^	0.1074	4.32					
	NaOH^∼^	1.5						
	KOH^∼^	1.75						
G6P3	G_6_P	0.0215	2.770	20	27.5	3.35	7.78	6.98
	NADP^+^	0.0574	2.73					
	NaOH^∼^	2.75						
	KOH^∼^	1.75						
G6P4	G_6_P	0.0213	2.80	20	27.0	3.31	7.82	6.99
	NADP^+^	0.0574	2.78					
	NaOH^∼^	3.1						
	KOH^∼^	1.5						
*CKase + ATPase* ^ø^								
*sequenced*								
CKATP_seq1_	CrP	0.0444	5.04	70	26.2	4.81	7.35	9.36
	ADP	0.0520	4.56					
	NaOH^∼^	0.8 mL						
	KOH^∼^	0.4						
*@ 20 *min *added*								
	ATP^χ^	0.0513	3.46^∴^	3	2.7	6.79	9.16	8.86
	NaOH^∼^	0.5						
	KOH^∼^	0.7 mL						
*simultaneous*								
CKATP_sim1_	CrP	0.0826	10.14	CK = 60	24.96	4.71	8.31	9.59
	ADP	0.0544	5.10	ATPase = 2.5				
	ATP	0.0635	5.02					
	NaOH^∼^	2.0 mL						
	KOH^∼^	0.6 mL						
	HCl	0.05 mL						
*sequenced*								
CKCrPATP_seq1_	Cr	0.027	5.13	50	27.05	3.98	9.27	8.02
	ATP	0.1317	9.60					
	NaOH^∼^	2 mL						
	KOH^∼^	1 mL						
*@ 10 *min *added*								
	ATPase	0.2 mL		2.5			7.93	7.56
*sequenced*								
CKCrPATP_seq2_	Cr	0.02014	4.65	40	29.0	3.93	9.23	9.04
	ATP	0.1355	9.21					
	NaOH^∼^	2.0 mL						
	KOH^∼^	1.0 mL						
*@ 10 *min *added*								
	ATPase	0.2 mL		2.5			9.03	8.21
*CKase + HKase + G*_*6*_ *PDH*								
*sequenced*				100 μL				
CKHKG6_seq_	CrP	0.0300	2.34	CK = 60	32.25	4.13	7.62	8.51
	Mg-ADP	0.0393	2.34					
	Mg-ATP	0.0154	0.77					
	Glucose	0.0168	2.37					
	G_6_P	0.0097	0.88					
	NADP^+^	0.0702	2.34					
	MgCl_2_^∼^	1.5 mL						
	dH_2_O	26.5 mL						
	NaOH^∼^	1.75 mL						
	KOH^∼^	2.5 mL						
*@ 10 *min *added*				HKase = 20			8.50	7.56
*@ 20 *min *added*				G_6_PDH = 20			7.56	6.88
*simultaneous*				100 μL				
CKHKG6_sim_	CrP	0.0253	2.84	CK = 60	27.21	4.12	8.11	6.92
	Mg-ADP	0.0323	2.78	HKase = 20				
	Mg-ATP	0.0127	0.92	G_6_PDH = 20				
	Glucose	0.0143	2.92					
	G_6_P	0.0071	0.92					
	NADP^+^	0.0584	2.80					
	MgCl_2_^∼^	1.25 mL						
	dH_2_O	22 mL						
	NaOH^∼^	1.56 mL						
	KOH^∼^	2.4 mL						

In order of completion: * including added chemicals for enzyme activation (see “Methods”) where Na^+^ was added as 0.1 N NaOH to contribute to adjusting pH_i_ to pH_start_; ^**^ stopped at 10 min; ^ volume adjusted for acid/base addition for pH_start_; (^#^) = pH at start of reaction, prior to addition of the enzyme; pH_end_ = pH at 20 min unless indicated otherwise; ^χ^ in 1.5 mL dH_2_O; ^∼^ from 0.1 M stock; ` from 0.02 M stock; ^ø^ Na^+^ and K^+^ needed for ATPase activation; ∴ based on the final volume of 29.22 mL; ^°^ original volume was 30.75 mL, but 3 mL was removed for latter spectrophotometric assays. Thus, substrate concentrations were calculated based on the 30.75 mL original reagent volume.

## Materials and methods

The methods of this research are explained in the order of their completion. The details of the chemicals and enzymes used for the study of the chemical reactions in Table 1 are presented in Table 2. As shown, all chemicals and enzymes were purchased from MERCK KGaA (Darmsadt, Germany). The required weights of all chemicals were calculated based on the supplier’s stated molecular weights, where final concentrations of metabolites were adjusted for reagent volume (∼40 or ∼25 mL) and slightly modified by the addition of necessary base and/or acid solutions. All reagents were prepared using deionized water (dH_2_O), along with any necessary enzyme activators (e.g., Na^+^ and K^+^ are necessary activators of porcine cerebellum Na^+^,K^+^-ATPase), substrate stabilizers (e.g., MgCl_2_ for ATP stability), and [Substrate] for different reagent conditions.

Table 3 presents the [Substrate], enzyme activities, reagent volumes (all reagents were developed using deionized water (dH_2_O)), and initial, start, and final pH values used to quantify (and resulting from) the pH changes for repeated trials of each reaction for slightly different conditions of all previously stated independent variables of the chemical reactions. The peak product accumulation ([Product]) for the selected reaction condition of each of the five chemical reactions is also provided. The [Substrate] ranged from 0.9 to 14 mmol ⋅L^-1^, and enzyme activities (total U for the reagent volumes) ranged from 2 to 100 International Units (U) (Table 3). All presented [Substrate] of Table 3 were corrected (from acid or base solution additions) for final reagent volumes for the targeted pH_start_ values (see below). All commercial lyophilized, unbuffered preparations of each enzyme were diluted by the addition of dH_2_O (e.g., 1,000 U lyophilized powder of known total U activity, mixed with 1 mL dH_2_O, where 10 μL = 10 U), stored frozen at −20 °C, and were used as stock enzyme solutions after thawing to room temperature.

Note that the pH conditions involved in this research extend beyond the physiological range in either direction. This was essential in order to quantify the magnitude of the H^+^_e_ relative to the product formation and resulting pH. To understand how a reaction impacts *in vivo* pH regulation, it is necessary to know the capacity of the reaction for altering pH. This can only be done using an *in vitro* model, which has been the standard research methodology for early inquiry into the biochemical details of individual chemical reactions and their subsequent roles in a cell-based complex system (Lehninger, 1960; 1961). To quantify the influence of a chemical reaction to pH, an unbuffered reagent is needed. As such, this initial inquiry could only be performed with the current methodology. Where dissociation constants were provided, these were obtained from research-based data bases (IUPAC Stability Constants Database; NIST Critically Selected Stability Constants) and prior research that used or contributed to these resources (LoPresti and Cohn, 1989; Golding et al., 1995; Kushmerick, 1997; Le et al., 2011; Robergs, 2017; Wiseman et al., 2023). The available dissociation constants and methodology were scrutinized to ascertain those based on temperature and ionic strength (I) conditions pertinent to cellular biochemistry and our conditions of *in vitro* measurements ((T∼25 °C); I∼200 to 300 mEq.L^-1^).

For the detailed procedure for the calibration or checking of the pH meter (pH50 Lab pH Meter, Westlab, Ballarat, Victoria, Australia), see Robergs et al. (2026). In short, known pH calibration standards at pH = 4.0, 7.0, and 10.0 were used to calibrate a maintenance free, open pore, Ag/AgCl glass polymer electrode with an integrated temperature sensor (CHS Polymer Green Temp (NTC30), Code CHS-5670-T1B; Chromservis, Petrovice, Czech Republic) allowing automatic pH adjustment to 25 °C. The pH meter was calibrated weekly or whenever initial checking of the electrode for each data acquisition session revealed deviations greater than 0.05 pH units from expected values for more than one calibration standard (pH = 4.0, 7.0, and 10.0).

The chemical reactions were based on a target reagent volume of either 40 or 25 mL. As will be explained, this was a targeted volume, as the extent of base (0.1 N NaOH and/or KOH) or acid (0.1 N HCl) addition to the reagent depended upon the specific reaction initial pH and target pH conditions; these, in turn, were also influenced by the [Substrate] and reagent volume. During the preparation of the reagents, the pH of the dH_2_O was measured and recorded so that the initial pH of the reagent could be better interpreted.

Once the reagent was made, the reagent beaker was placed on a previously heated magnetic stirrer to cause and retain a temperature as close as possible to 25 °C (widest range ∼23 °C–27 °C), and the pH electrode was placed in the beaker; see Robergs et al. (2026) for further details. Once the reagent pH stabilized, the value was recorded as the initial pH (pH_i_) reading of the reagent. The necessary volumes of NaOH and KOH occurred to attain targeted pH values (pH_start_) via known volume additions of 0.1 M stock solutions, with further addition of the necessary volumes of 0.1 N HCl if minor corrections to pH were required. The added acid or base volumes were recorded, and once a stable pH was established that was close to the targeted value, the final adjusted pH value was recorded.

These desired pH_start_ values differed depending on the expected pH change for each reaction direction (16) and the [Substrate]. The ATPase reaction was only studied in the direction of ADP production, where it was expected to lower pH (Kushmerick, 1997; Vinnakota et al., 2006; Robergs, 2017). The pH_start_ values were manipulated to be close to pH = 9.0, though for added trials, pH_start_ values were also studied below pH 9.0 to document the influence of inorganic phosphate (HPO_4_^−2^) production to pH-dependent H^+^ buffering (pKa = 6.75; HPO_4_^−2^ + H^+^ ↔ H_2_PO_4_^−1^) (Robergs, 2017).

The creatine kinase reaction was expected to raise pH in the direction of ATP production but lower pH in the direction of CrP production (Kushmerick, 1997; Vinnakota et al., 2006; Robergs, 2017). Both the HKase and G_6_PDHase reactions are highly exergonic and hence effectively unidirectional, and both cause the release of a H^+^ (Kushmerick, 1997; Vinnakotia et al., 2006; Robergs, 2017; Voet and Voet, 2018; Nelson and Cox, 2021). As such, pH_start_ values were always close to or above 8.0 for these reactions. There was variability in attaining targeted start pH values for repeated pH profile data acquisitions based on the difficulties in developing a reagent without added H^+^ buffers.

Once the reagent attained a stable pH_start_ value, the baseline pH was recorded every 15 s for 2 min to document stability. To assist in accurate data collection, video recording was then started, a digital timer was reset, and the chemical reaction was commenced by the addition of the required volume of known enzyme standard solution (typically 10–100 μL, depending on the reaction, enzyme activity, and enzyme cost and availability). The digital timer was then re-started (<2 s delay), and the pH readings were manually recorded as follows: every 10 s from 0 to 2 min, every 20 s from 2 to 5 min, every 30 s from 5 to 10 min, then every 1 min from 10 to 20 min.

For the reactions from which product measurements were intended (CKATP7, CKCrP7, ATP7, HK3, and G6P2; see Table 3), 200 μL samples of the reagent were dispensed into vials containing 100 μL of 0.1 M HCl, followed immediately by brief vortexing (∼2 s), the addition of 30 μL of 0.1 M KOH, followed by the addition on 20 μL of 0.5 M Tris base and a final vortex (total time for the entire sample method approximated 35 s). Prior pH measurements of this method revealed that the combined KOH and Tris base raised the pH of the solution of HCl-plus reagent sample to 7.65. Samples were obtained at baseline, 10 s, then every 30 s to 1 min 40 s, then every 40 s to 5 min, then every 1 min to 10 min, then every 2 min to 20 min. All sample specimens treated with base and buffer addition were assayed immediately after the pH profile data collection as described below.

After the completion of the 20 min data recording of the pH changes during the chemical reaction, all acidified then pH neutralized sample vials were placed in the refrigerator at 4 °C during the preparation of the assay reagent and test tube labelling (∼40 min). Once the reagents were prepared, the reagent samples were retrieved from fridge storage. Each sample was vortex mixed, and then 100 μL of the sample was added to 1 mL of a reagent in borosilicate test tubes in duplicate. This was repeated in sequence for each sample. The reagent differed depending on the reaction and the product of interest; these details are presented in Table 4. In short, the reagents for all product assays were developed to result in a stoichiometric increase in NADPH for the product of interest (Lowry and Passeneau, 1972; De Ruyck et al., 2007). This involved the sequential linking of the CK reaction to ATP production to the HKase reaction, which was then linked to the G_6_PDHase reaction (see Table 1). For the CKase reaction in the direction of CrP production, the decrease in ATP was quantified as the stoichiometric increase in CrP via the same sequenced HKase and G_6_PDHase reagent. For the HKase reaction to G_6_P production, G_6_P was the product of interest, which was linked to the G_6_PDHase reaction. For the G_6_PDHase reaction to 6PGl, the increase in NADPH was equated to the decrease in G_6_P and stoichiometric increase in 6PGl production. Such assays were performed with H^+^ buffering (Tris base; Table 3), combined with controlling the pH_start_ of the reagent to either slightly acidic or moderately alkaline values (depending on the chemical reaction; Table 4).

**TABLE 4:** Composition of chemical reagents used to complete the test-tube assays for specific products of the chemical reactions of interest during pH-to-time profile trials.

Ingredients	Reaction product reagents				
	CKase → ↑ATP	CKase → ↓ATP	ATPase → ↓ATP	HKase → ↑G_6_P	G_6_PDHase → ↓G_6_P
Glucose	3.0	3.0	3.0		
NADP^+^	3.0	3.0	3.0	3.0	3.0
Tris base	2.5	2.5	2.5	2.5	2.5
MgCl^^^	3 mL	3 mL	3 mL		
dH_2_O	47 mL	47 mL	47 mL	49 mL	49 mL
NaOH^*^	1.5 mL	1.5 mL	1.5 mL		
HCl^*^				0.75 mL	0.4 mL
HKase^∼^	25 μL	25 μL	25 μL		
G_6_PDH^∼^	25 μL	25 μL	25 μL	25 μL	30 μL
Total volume	51.55 mL	51.55 mL	51.55 mL	49.78 mL	49.70 mL
pH	6.57	6.57	6.57	8.95	9.03

All chemical concentrations expressed in mM.L^-1^; ^*^ From 0.1 M stock solution; ^From 0.02 M stock solution; ^∼^ From stock solutions of 10 μL = 10 U.

As with prior research of the LDH reaction (Robergs et al., 2026), to establish added evidence and accuracy for the magnitude of product formation, a cuvette spectrophotometric method was developed to repeat the targeted chemical reaction. The details of these reagents are provided in Table 5. In summary, the reagents required the presence of substrates and enzyme co-factors. The addition of the enzyme(s) instigated the start of the chemical reaction and always occurred 2 min into data acquisition. However, due to the function of ATP as a substrate in the CKase reaction to CrP and to the ATPase reaction, these reactions could not be studied by this direct spectrophotometric method. In contrast to the test-tube reagent development, the involvement of H^+^ buffers was not required, as the reagent pH was adjusted to alkaline values prior to data acquisition. The added feature for the cuvette reactions was that a large dilution of the reagent was necessary (and hence very low absolute product formation and related pH challenge) to allow valid absorbance measures for the constrained absorbance range of the spectrophotometer (∼3.2 units) (see Table 5).

**TABLE 5:** Composition of chemical reagents used in the direct cuvette assay of the kinetic changes in [NADPH] linked to the product formation of the chemical reactions of interest using enzymatic spectrophotometry.

Component and condition	Unbuffered reaction reagents		
	CKase → ATP	HKase → G_6_P	G_6_PDHase → 6PGl
Glucose	3.0	3.57	
G_6_P			2.86
CrP	3.0		
ATP		2.78	
ADP	3.0		
NADP^+^	3.0	5.78	3.02
MgCl	0.5 mL	0.35 mL	
dH_2_O	9.5 mL	4.5 mL	4.2 mL
NaOH	200 μL	350 μL	400 μL
KOH	500 μL	400 μL	600 μL
CKase	20 U		
HKase	10 U	2 U	
G_6_PDH	10 U	2 U	5 U
Total volume	10.24 mL	5.6 mL	5.2 mL
pH	9.43	8.65	8.96
Dilution assays	x3; x4; x5	x5; x10	x5; x6.6667

All chemical concentrations expressed in mmol L^-1^.

Based on past completions of the different chemical reaction pH to time profiles, in addition to an assessment of remaining chemical supplies relative to the reality of commercial access to needed chemicals, given conditions of [S], pH_start_, and enzyme activity were chosen to quantify test-retest reliability and validity. The conditions for these added trials are presented in Table 3 for the two reactions of HKase and G_6_PDHase. The CKase reactions are not included due to the inability to purchase added stock of the enzyme. Study of the ATPase reaction was prevented based on cost and the low enzyme activity (5 U) of the available product. All reagents used for the duplicate pH-to-time profiles were made separately, and each paired set of pH-to-time profiles of a given reaction occurred sequentially on the same day, with approximately 45 min separating each trial due to subsequent reagent preparation, pH measurement, adjustment of the reagent as needed, and pH meter calibration. The HKase reactions occurred on 1 day, followed by the G_6_PDHase reaction the next day.

To explore the implications of the direct release or consumption of H^+^ during chemical reaction H^+^_e_ to the pH of the aqueous reagent, and in addition to the role of phosphate group H^+^ buffering (H^+^ + HPO_4_^−2^ ↔ H_2_PO_4_^−1^) to the pH of the reagent during the ATPase reaction, the modified expression of the Henderson–Hasselbach equation derived by Robergs et al. (2026) was used (Equation 1) for reactions that release a H^+^. It is acknowledged that this approach is unlikely to accurately depict the expected pH results for the chemical reactions. As such, it is more of a computed comparison to establish the relevance of additional factors that influence the pH responses. Based on prior computational chemistry research of similar chemical reactions (Kushmerick, 1997; Vinnakota et al., 2006; Robergs, 2017), additional adjustments would involve the pH-dependent H^+^ association of the remaining [Substrates] and [Products] at the specific times of the pH profile data acquisitions, the enzyme, in addition to as yet unclear involvement of the influence of the reagent ionic strength and water ionization to the pH responses to chemical reaction H^+^_e_ (see “Conclusions and Recommendations”).

$$pH=-\log\left[Product\right].$$ (1)

### Data processing

All data were entered into a commercial spreadsheet program (Excel, Microsoft Corporation, Redmond, WA, USA), followed by importation into a commercial curve fitting and graphics program (Prism, GraphPad Software, Boston, MA, USA). All pH data were converted to [H^+^] and expressed as M × 10^9^, where pH = 7 equates to a [H^+^] = 100 × 10^−9^ M). All pH- and [H^+^]-time profiles and product formation-time profiles were processed for best fit (lowest standard error of the estimate; SE) linear or non-linear functions. To derive data for the pH versus [Product] and [H^+^] versus [Product] profiles, the linear or non-linear equations and their resulting coefficients for pH versus time, [H^+^] versus time, and [Product] versus time were programmed into custom software (LabVIEW™, v-2017, National Instruments, Austin, TX, USA), and data were generated at 1 Hz across the 20 min of each reaction data collection, providing 1201 data points for each profile. These results for pH and [Product] were compiled into two-dimensional arrays and then saved as .txt files for subsequent importation into the commercial curve fitting and graphics program.

The test’s re-test reliability was performed using commercial graphics and statistical software (Prism, GraphPad Software, Boston, MA, USA). Not all data points (n = 42) were used for each linear regression of each reaction. This was due to the need to remove the initial data points (two to six, depending on the reaction) if the pH change was rapid and revealed the poor temporal resolution of the pH meter. The end data points of each reaction also needed to be screened and removed after the stable peak pH had been obtained and/or pH values slowly reversed direction. Such procedures will be become clearer from the figure data presented in “Results” below.

## Results

### Single reaction pH profiles

The data for the pH profiles are presented in Figure 2 for a) CKase to ATP, b) CKase to CrP, c) ATPase, d) HKase, and e) G_6_PDH. For the CKase-to-ATP reaction, pH increased, and for the reverse direction of the reaction (to CrP production), it decreased. Such pH changes are in accord with the organic chemistry of the chemical reactions and adhere to prior computation chemistry evidence (Table 1) (Kushmerick, 1997; Vinnakota et al., 2006; Robergs, 2017; 2019; 2021). The variability in the pH profiles occurred due to a mix of variability in the [Substrate] and enzyme activities, pH_start_, and more importantly, the deteriorating catalytic activity of the CKase after repeated freezing and thawing. This is most notable for CKATP6, CKATP7, and CKCrP1, which were all acquired using relatively “fresh” enzyme. As with our prior research results (Robergs et al., 2026) and to reinforce the content presented in “Methods,” the range of pH responses for this research is large and non-physiological. This is to be expected in order to document the capacity of the H^+^ removal or release of these chemical reactions. As such, we used pH as a dependent (response), not a controlled, measure. Once this reaction specific capacity for pH change relative to product formation is known, the role of these reactions in the regulation of pH within the physiological pH range can be better understood (see “Discussion”).

**FIGURE 2:**
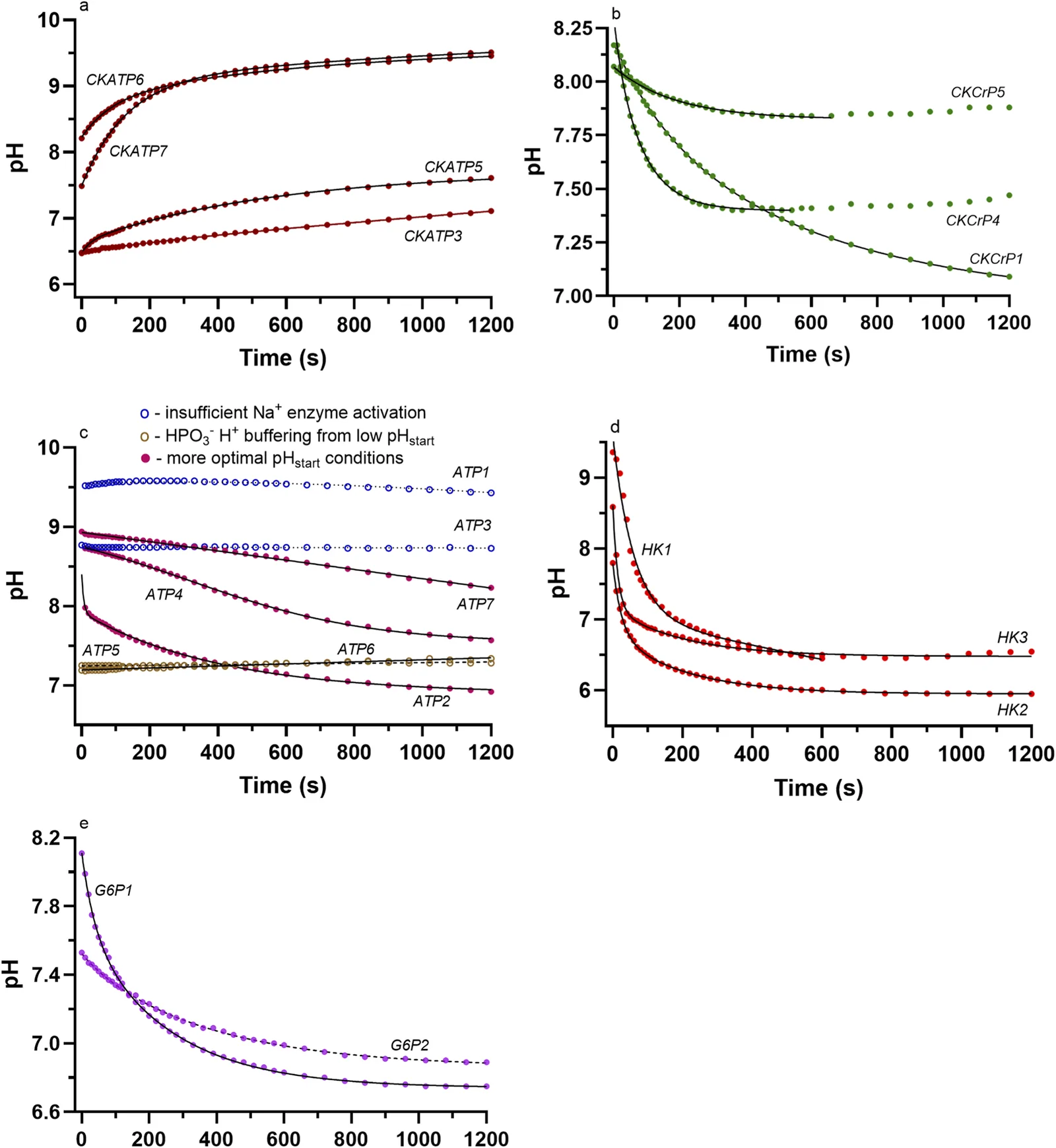
Selection of pH-to-time profiles of 20 min chemical reactions of **(a)** CKase to ATP, **(b)** CKase to CrP, **(c)** ATPase to ADP, **(d)** HKase to G_6_P, and **(e)** G_6_PDH to NADPH. Reaction codes used to identify specific reaction conditions as presented in Table 3.

For Figure 2c, the ATPase reaction to ADP is a complex reaction, as the ATPase used (Table 2) was from the ATPase-linked Na^+^-K^+^ pump of porcine cerebellum (Na^+^,K^+^-ATPase) (Table 2). Such an isozyme is regulated by Na^+^ and K^+^ (both essential activators), which is evident from the blunted pH profiles of ATP1 and ATP3, which had none-to-minimal Na^+^ in the reagent. As the ATPase reaction also produces HPO_4_^−2^, which is a H^+^ buffer of physiological relevance (pK = 6.75), the pH_start_ used in this reaction pH profiling is also important due to its influence on the pH-dependent pH response of the chemical reaction. Such H^+^ buffering for HPO_4_^−2^ to H_2_PO_4_^−1^ increases as pH decreases from ∼8.25 towards the pK value and lower, where the effective buffering range is approximately 1.5 pH units above to below the pK. It is for these reasons that most investigation of the pH profiles of the ATPase reaction to ADP have pH_start_ values above 8.5 (Table 3; Figures 2c, 5e). Consequently, the kinetics of the pH changes are slower than for each of the other reactions, which, depending on the pH, is a combination of the H^+^ buffering from HPO_4_^−2^ and use of relatively low enzyme activities of the ATPase due to the expense of this enzyme. This is observed in the blunted pH profiles of ATP4, ATP5, and ATP6.

Despite these observations and considerations, it is clear from the data that the ATPase reaction lowers the pH of the reagent due to the release of a H^+^ as governed by the organic chemistry and computational chemistry of the chemical reaction (Table 1). However, as noted by Robergs (2017), the pH dependence of the H^+^_e_ of this reaction is incredibly important, and across the physiological cellular pH range, the net H^+^_e_ release of this reaction diminishes (though remaining negative = release) as pH decreases below 7.0. Such a pH response from this research is more impressive considering the low enzyme activity conditions that were used (2–5 U). As mentioned, this was necessitated by the expense of the Na^+^,K^+^-ATPase enzyme. For comparable Na^+^,K^+^-ATPase enzyme activity to the CK reaction, the pH response to the ATPase reaction would have been much larger (as product formation would have been larger) for pH ranges above the H^+^ buffering range of HPO_4_^−2^ (6.75 ± 1.5 pH units).

The HKase reaction (Figure 2d) is a phosphate transfer reaction (Table 1) that is potent in the rapidity and magnitude of the resulting decrease in the pH of the reagent. Such a response is similar to the G_6_PDHase reaction, which is an oxidation–reduction reaction, similar to that of the LDHase reaction (Robergs et al., 2026). As for the LDHase reaction in the direction of pyruvate production (oxidation of lactate), the G_6_PDHase reaction causes the release of a H^+^ to the reagent during the oxidation of G_6_P, thereby causing a dramatic decrease in reagent pH. Of the five reactions, the HKase reaction had the largest and most rapid pH change; as will be revealed, this was linked to a relatively large [Product]. It is also worth noting the relatively large H^+^ coefficient for the HKase reaction for pH conditions of 7 and 6, being −1.258 and −1.850, respectively (Table 1) (see “Discussion”).

### Test–retest reliability and validity

As multiple trials were not completed for each [Substrate], pH_start_, or enzyme activity conditions of the chemical reactions, the alternative means of documenting the reproducibility of the pH-to-time profiles of the chemical reactions was to perform replicate trials for each reaction of interest. Such replication enabled the computation of the reliability coefficient of the two trials of each reaction (the consistency of the relative change in pH from one trial to another), the standard error of estimate (S_y.x_) of the two trials indirectly revealing the accuracy (validity = lower residuals error) of the duplicate pH-to-time profiles. As explained in “Methods,” such procedures were confined to the HKase and G_6_PDH reactions due to the commercial unavailability of additional CKase and the expense of the Na^+^,K^+^-ATPase enzyme. The results are also an extension of the results of Robergs et al. (2026) for the D- and L-LDHase reactions to each lactate and pyruvate, where all reactions also had high reliability coefficients (*R* ^2^ > 0.99).

The data of the test–retest trials are presented in Figures 3a,b for HKase and Figure 3c,d for G_6_PDH. The reaction conditions for the i- and ii- HK reactions are for HK4 and 5, respectively (see Table 3). The reaction conditions for the i- and ii- G_6_PDHase reactions are for G6P3 and 4, respectively (see Table 3). The [H^+^] data for each of the HKase and G6PDH reactions are not presented, though based on mathematical logic, the results are of similar precision. The regression statistical results for both of pH and [H^+^] follow. Statistical results are presented for the regression equations, *R* ^2^, S_y.x_, F, df, and *p*-value for the pH and [H^+^] for each reaction. Results for the HK-reaction pH data were y = 1.055a – 0.3754, 0.9971, 0.01127, 5894, 1:25, *p* < 0.0001. Results for the [H^+^] data were y = 1.049a – 4.723, 0.9963, 1.485, 6388, 1:24, *p* < 0.0001. Results for the G_6_PDHase pH data were y = 1.069a – 0.5452, 0.9984, 0.007879, 14,563, 1:24, *p* < 0.0001. Results for the [H^+^] data were y = 1.158–2.812, 0.9976, 1.178, 10,653, 1:26, *p* < 0.0001. Note that the [H^+^] data for S_y.x_ are larger than the pH data simply because the [H^+^] data were expressed as M × 10^9^ as explained in “Methods (*Data Processing*)” and identified in all pertinent figures.

**FIGURE 3:**
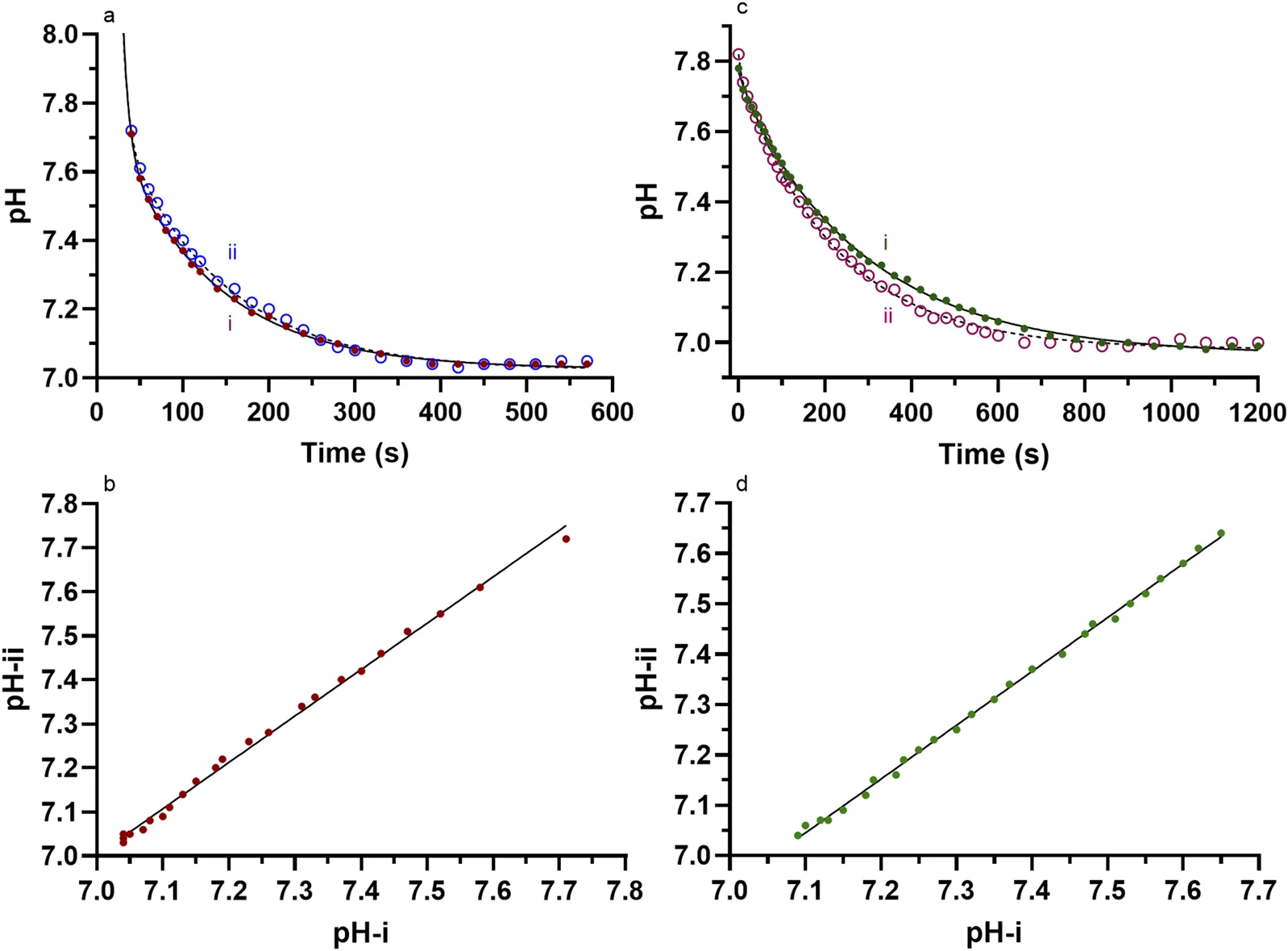
The data of the test-retest reliability and validity for the replicate trials (coloured closed and open circles; i=·, ii=O) of the pH-time profiles for **(a)**) the HKase reaction, where the data sets of Table 3 are HK4 (i) and HK5 (ii), respectively. The resultant linear regression of both pH data sets is presented in **(b)**. The data for the same conditions of the G_6_PDH reaction are presented in **(c,d)**, respectively, where the data sets of Table 3 are G_6_P3 (i) and 4 (ii), respectively. See text for the statistical results of the test, re-test reliability.

### Sequenced and simultaneous reactions

Given the results of the individual chemical reactions, there was interest in whether such pH responses behave similarly when chemical reactions causing similar or opposing pH responses are combined in sequence or simultaneously. To determine this, initially the CKase reaction to CrP was sequenced with the ATPase reaction (Figure 4). As shown, the addition of the H^+^ release of the ATPase reaction caused a more rapid decrease in the reagent pH, as predicted by the organic and computational chemistry. Consequently, the chemical reaction H^+^_e_ remains relevant in the presence of other chemical reactions; such pH responses are quantum in regard to the net (combined) influence of the multiple reactions to the reagent pH and immediate regarding the non-erratic nature of the pH responses. This latter observation has relevance to Mitchell’s proticity concept (Mitchell, 1976) (see “Discussion”), and especially when applied to *in vivo* conditions and their related alterations to H^+^_e_ and aH_3_O^+^ that are linked through enzyme complexes. Such results stimulated further interest in other examples of reaction combinations, which are presented in Figures 5, 6.

**FIGURE 4:**
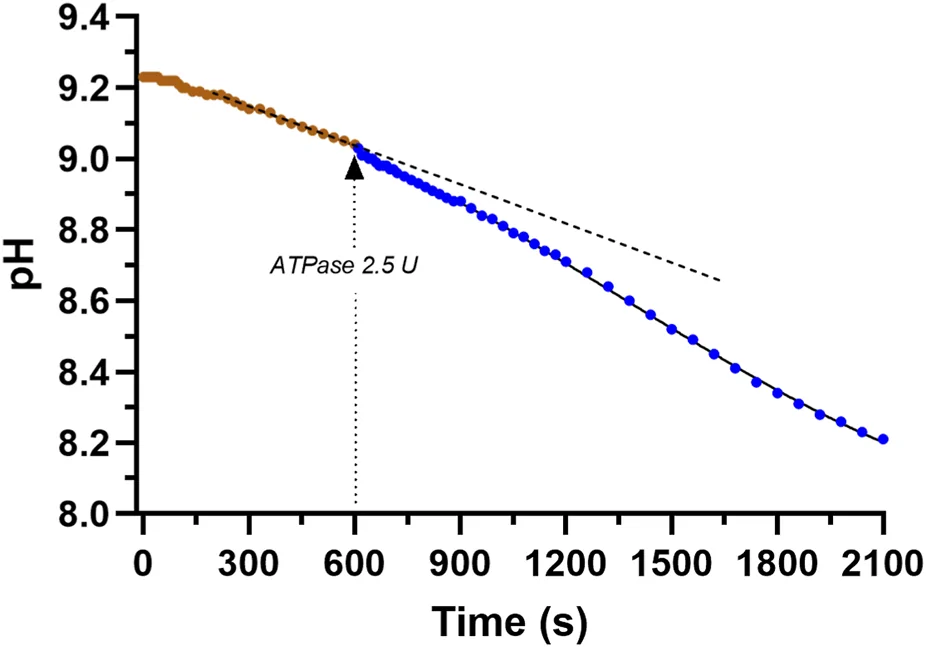
pH profiles for CKase-to-CrP and -ATPase reactions (CKCrPATP2 of Table 3), where the CK reaction occurred for the initial 600 s (10 min) followed by the additive activity of the ATPase reaction (600–2100 s; 10–35 min). Note the clear summative feature of the combined reactions after 10 min when pH is kept above 8.2.

**FIGURE 5:**
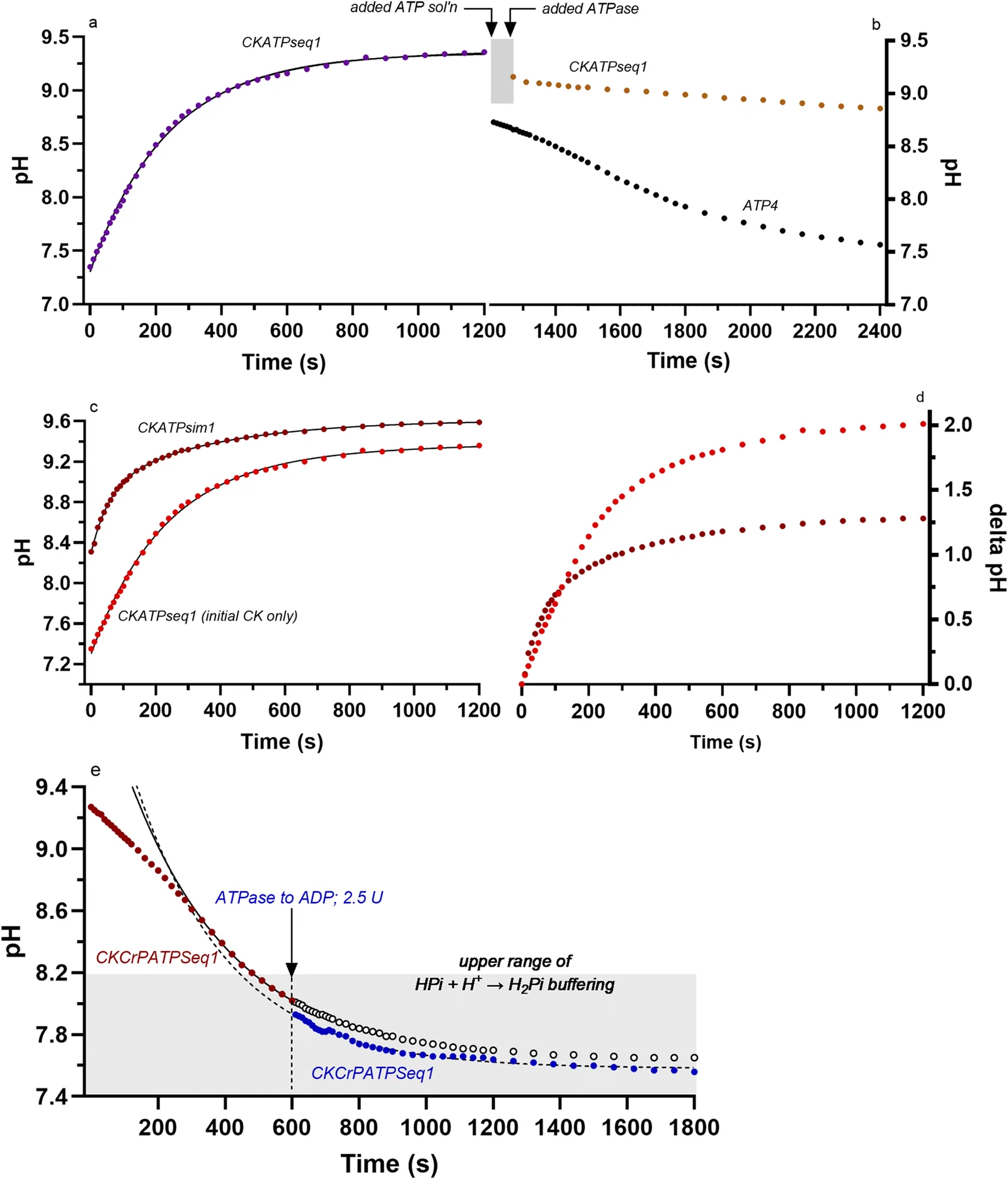
pH profiles for varied sequenced and simultaneous conditions of CK and ATPase reactions. **(a)** CK reaction to ATP, immediately followed by **(b)** addition of ATP solution (4 mM for 25 mL total volume) and then 2.5 U of ATPase, resulting in the pH profile of the ATPase sequenced reaction compared to the pH profile of the single ATPase reaction (black symbols). **(c)** pH profile of combined CK and ATPase reactions (brown symbols) compared to pH profile of the singular CKase reaction (red symbols = same data from Figures 4a, 6a). **(d)** Data of Figure 6c expressed as delta values from time = 0. **(e)** pH profile of 10 min of the CK reaction in the direction of CrP formation, followed by the addition of the Na^+^,K^+^-ATPase enzyme. The open symbols represent the computed continued mono-exponential decay profile of the CK reaction, revealing the minimal influence of the added H^+^ release of the ATPase reaction. Note gray range of the upper limit of the H^+^ buffering by HPO_4_^−2^ (to H_2_PO_4_^−1^) for pH values <8.2.

**FIGURE 6:**
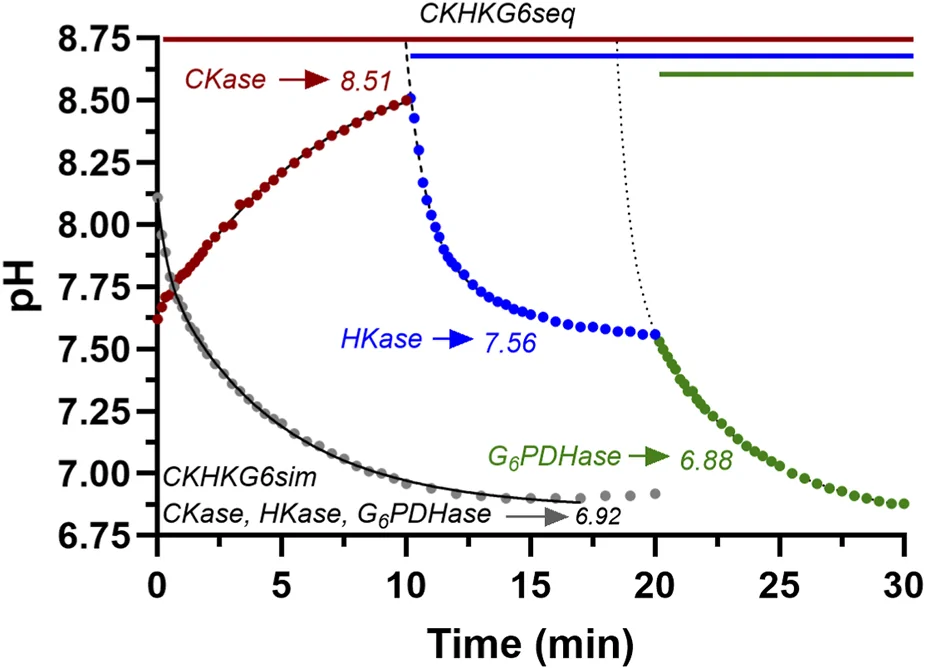
pH profile alterations for the CKase, HKase, and G_6_PDH reactions when analyzed sequentially (Table 3, CKase + HKase + G_6_PDH; brown, blue, and green data points, respectively) vs. simultaneously (Table 3, CKHKG6sim; grey data points). Note the summative (positive and negative) features of the pH responses.

The extent of the blunted pH response to the ATPase reaction while there is ongoing CKase reaction activity was further established when comparing responses after a longer duration for the CKase reaction (Figures 5a,b). For this instance, the direction of the CKase reaction was to ATP, and hence H^+^ consumption and an increase in pH. After 20 min, both the ATP substrate and the Na^+^,K^+^-ATPase enzyme were added. The extent of the CKase reaction causing a restrained pH decrease from the ATPase reaction is best seen when directly comparing it to the individual ATPase reaction pH response (Figure 5b, black symbol data). The pH response to simultaneous CKase and ATPase reactions is shown in Figures 5c,d, in comparison to the individual CKase to ATP reaction (red data points, dashed line). However, the pH response data comparison is complicated by the need for different pH_start_ conditions related to the HPO_4_^−2^ production of the ATPase reaction, as previously noted. When adjusting for the different pH_start_ values (Figure 5d), the differences in the pH responses are seen more clearly. Finally, the concern over the complication of HPO_4_^−2^ production of the ATPase reaction is verified once again by the data of Figure 5e, where there are blunted pH responses when the two reactions are combined across lower pH values.

It was logical to compare the pH responses without HPO_4_^−2^ buffering (no ATPase reaction) interference across the three main chemical reactions conducted in sequential order, where the CKase reaction to ATP would increase pH and where the pH responses of each of the HKase and G_6_PDHase reactions would lower pH. Such responses are seen in Figure 6, where the staircase effect is striking for all three reactions. Once again, such data is strong evidence for the quantum and independently consistent functions of each reaction to the overall pH response. The three sequenced reactions can then be compared to the three reactions that occur simultaneously (grey data as labelled) where the HKase and G_6_PDHase reactions dominate the pH response, though the delta magnitude of the collective pH response is less than when sequenced. In other words, the simultaneous condition demonstrates how the CKase reaction diminishes the magnitude of the decreases in pH from the HKase and G_6_PDHase reactions. Such evidence further reveals the likely simple quantum computational and near immediate (proticity) nature of the pH responses of simultaneous chemical reactions in more complex systems.

### Product formation

Data for the comparison of the pH-to-time, pH-to-[Product], and [H^+^]-to-[Product] profiles of the trials used for quantifying [Product] of the chemical reactions are presented for the CKase reaction to ATP (Figures 7a-c), CKase reaction to CrP (Figures 8a-c), ATPase reaction to ADP (Figures 9a-c), HKase reaction to G_6_P (Figures 10a-c), and the G_6_PDHase reaction to NADPH (Figures 11a-c). Interestingly, the [Product] increase for the CKase reaction to ATP was relatively high (close to 1.95 mmol·L^-1^) and much lower for the direction to CrP (0.64 mmol·L^-1^). Both directions of the CKase reaction caused a non-linear increase in [Product], though the kinetics of the two-phase association responses was much slower for the direction to CrP production (Figure 7b vs. 8b). The ATPase reaction, despite reagents having low enzyme activity and production of the physiologically relevant H^+^ buffer of HPO_4_^−2^, yielded a peak [Product] of 0.84 mmol·L^-1^. As is to be expected based on the previous explanation of low enzyme activity and HPO_4_^−2^ production, the kinetics were slow, which, in turn, explains the moderate [Product]. The HKase reaction had the largest [Product] at 2.36 mmol·L^-1^ (Figure 10b), with moderate kinetics. The G_6_PDHase reaction had a remarkably linear [Product]-to-time profile for the majority of the product and pH range, with a moderate peak [Product] of 1.46 mmol·L^-1^ (see Table 3 for reaction details).

**FIGURE 7:**
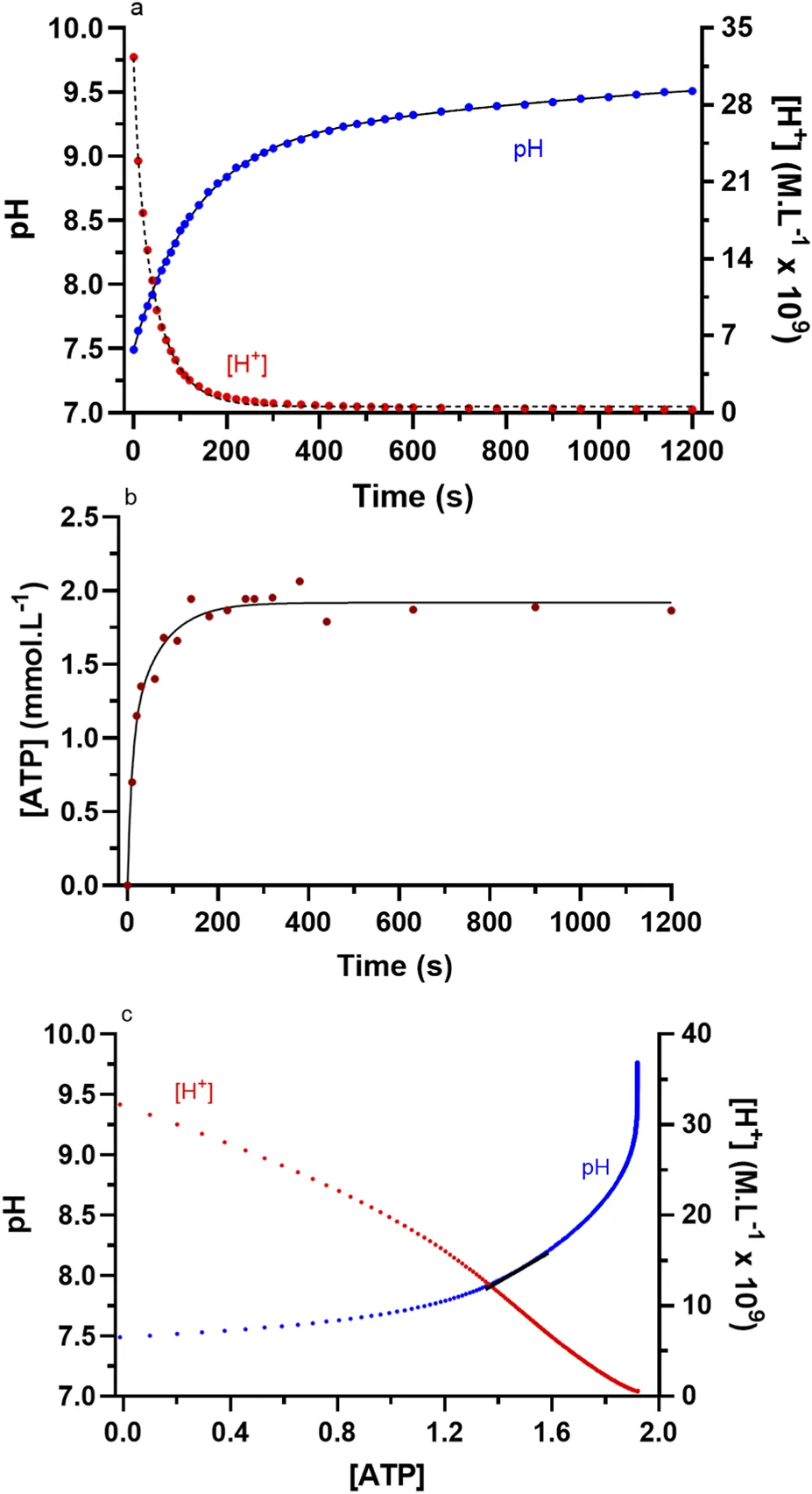
Complete data for CKase reaction to ATP production (CKATP7) for **(a)** pH and [H^+^] time profiles, **(b)** increase in ATP production across the 20 min reaction, and **(c)** computed pH and [H^+^] to [ATP] profiles of the chemical reaction.

**FIGURE 8:**
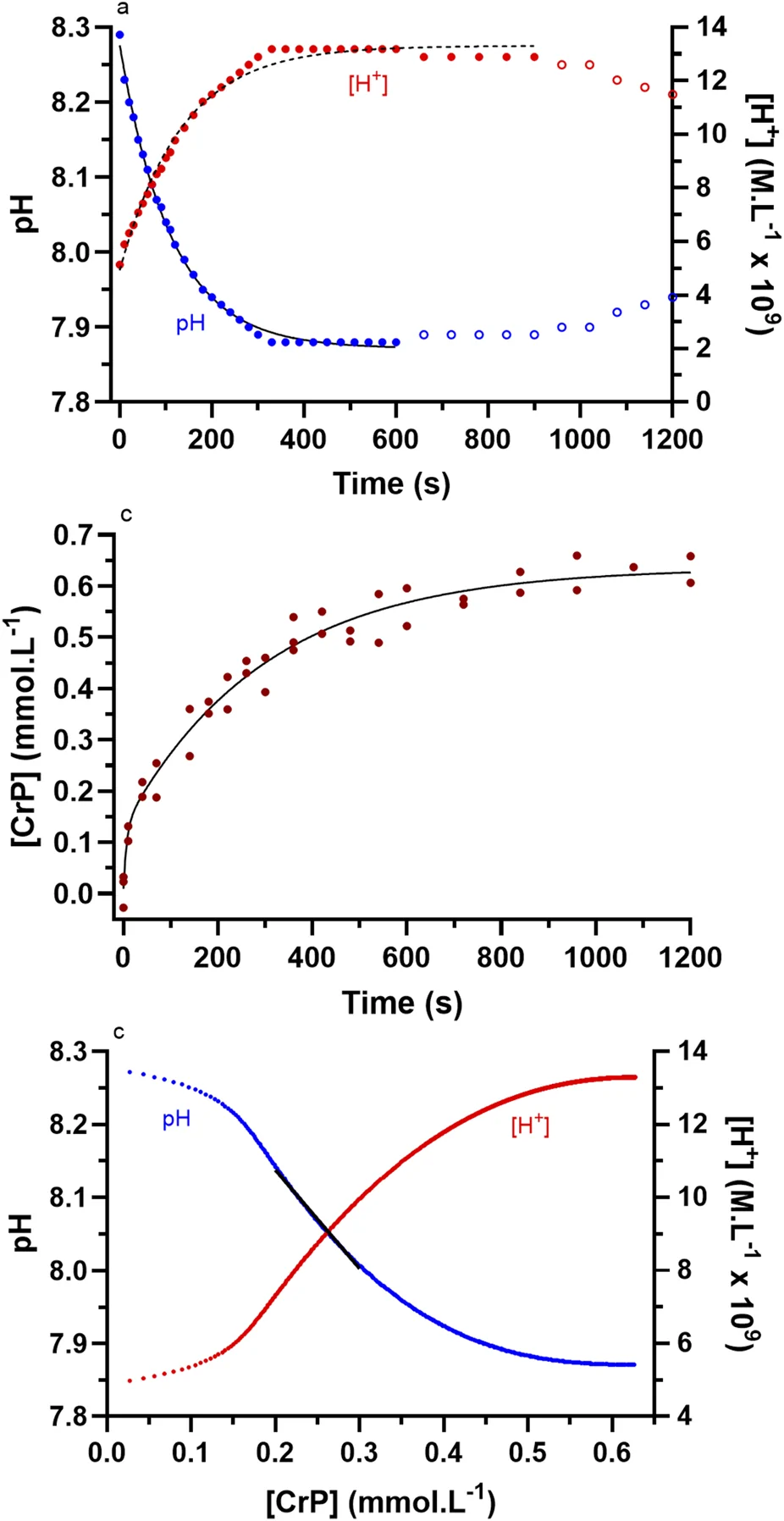
Complete data for the CKase reaction to CrP production (CKCrP7) for **(a)** pH and [H^+^] time profiles, **(b)** increase in CrP production across the 20 min reaction, and **(c)** computed pH- and [H^+^]-to-[CrP] profiles of the chemical reaction.

**FIGURE 9:**
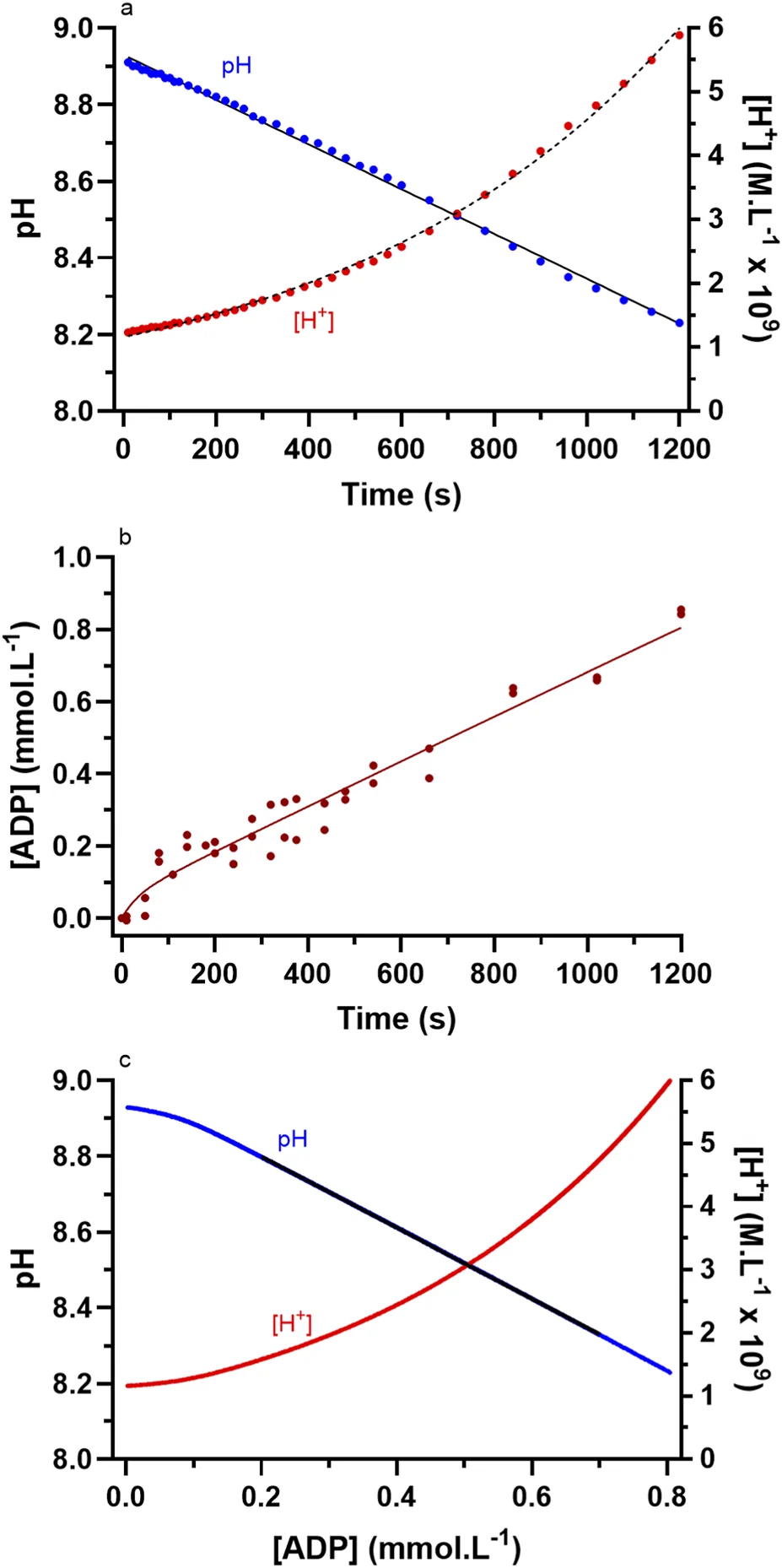
Complete data for the ATPase reaction (ATP7) for **(a)** pH and [H^+^] time profiles, **(b)** increase in ADP production across the 20 min reaction, and **(c)** computed pH- and [H^+^]-to-[ADP] profiles of the chemical reaction.

**FIGURE 10:**
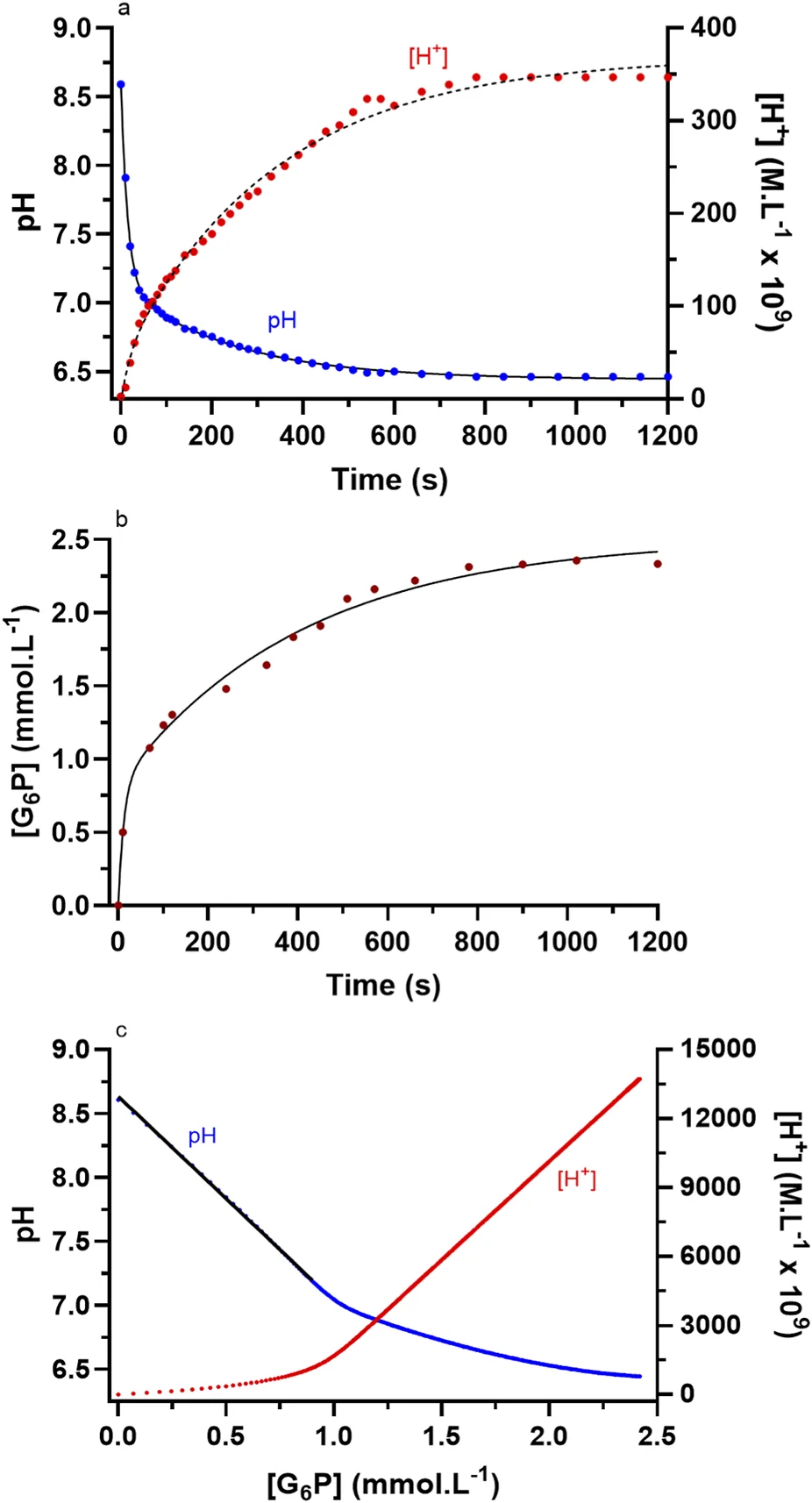
Complete data for the HKase reaction (HK3) for **(a)** pH and [H^+^] time profiles, **(b)** increase in G_6_P production across the 20 min reaction, and **(c)** computed pH- and [H^+^]-to-[G_6_P] profiles of the chemical reaction.

**FIGURE 11:**
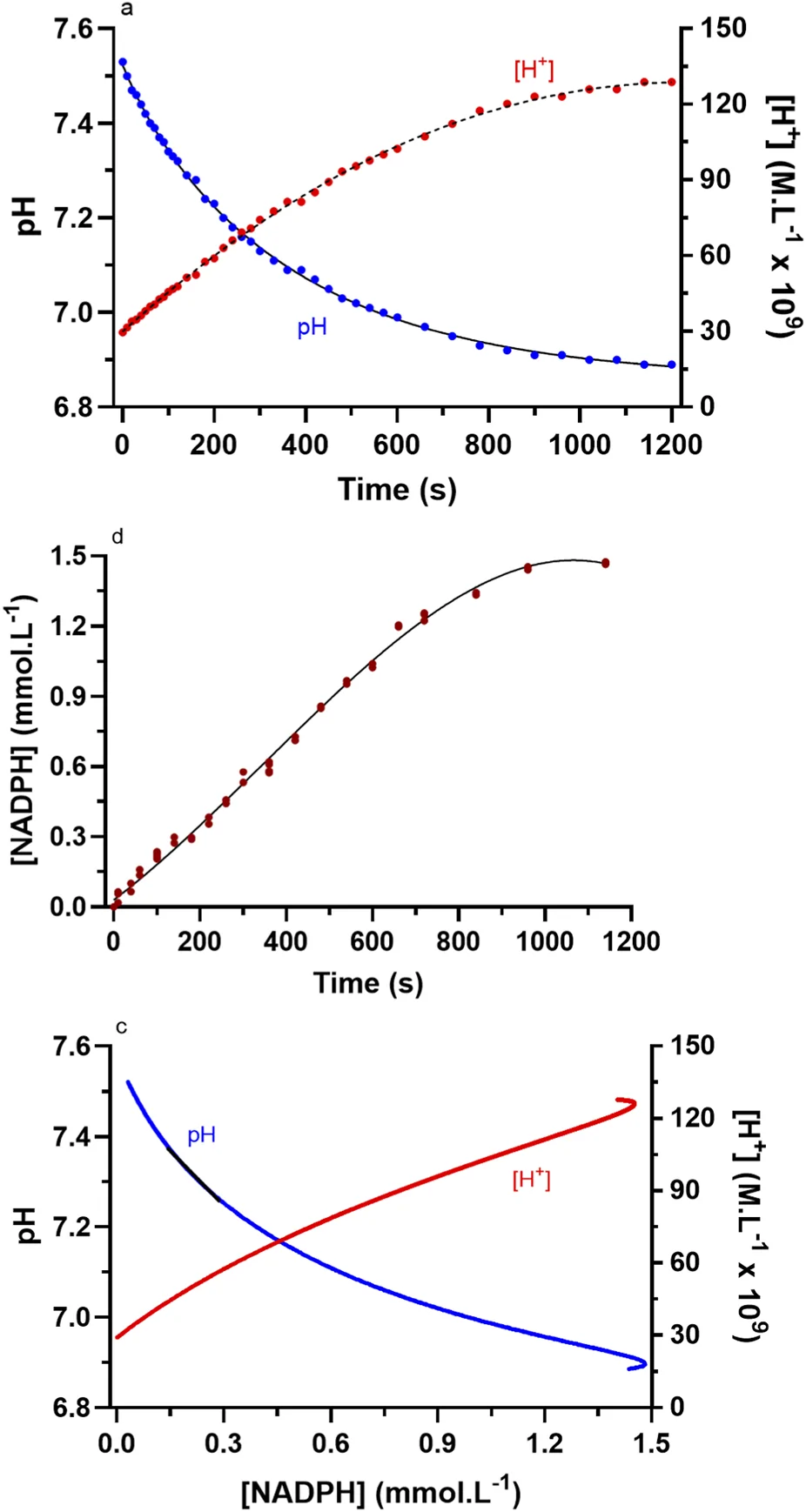
Complete data for the G_6_PDH reaction (G6P2) for **(a)** pH and [H^+^] time profiles, **(b)** increase in NADPH production across the 20 min reaction, and **(c)** computed pH- and [H^+^]-to-[NADPH] profiles of the chemical reaction.

The pH- and [H^+^]-to-[Product] profiles are presented in the c-subset figures of Figures 7–11. Such profiles for each reaction all have similarities and differences within each profile for pH and [H^+^]. As the pH unit is logarithmic, it would be expected that linear pH-to-[Product] profiles would yield curvilinear [H^+^]-to-[Product] profiles. It would also be expected that if the H^+^_e_ of chemical reactions is stoichiometric to [Product], there would be consistent pH or [H^+^] to [Product] profiles across the range of [Product]. As shown in each of Figures 7c, 8c, 9c, 10c, 11c, this was not the case. Conversely, given the wide range in pH for most chemical reactions, combined with the direct involvement of added H^+^ interacting with the natural ionization of water, it would also be logical to expect a variant [H^+^]-to-[Product] profile.

Despite the comments above, the results of the pH- and [H^+^]-to-[Product] data should be interpreted with caution. The limitation of this approach using our methodology is the difficulty in accurately profiling both pH and [Product], which can change rapidly over a narrow time range that often induces the majority of the pH and [Product] response in < 5 min. Targeted reactions with moderate [S] and enzyme activities were chosen to extend this time range of data change, but in prolonging the pH response and [Product] accumulation there is a lowering of the magnitude of both responses, which, in turn, lowers the sensitivity of the accurate measurement of both variables. These collective concerns are apparent for the CKase reaction to ATP, as the product formation was essentially complete within 3 min, as was the bulk of the pH response. The slower kinetics of each of the other reactions opens that data to more accurate interpretations of the association between pH change and product formation; such emphasis will be documented in the short descriptions of these results that follow.

The [Product] response for the ATPase reaction is uniquely linear compared to the other chemical reactions. This is likely caused by the low enzyme activities used in this research, which is inherently caused by the high expense and constrained availability of this product from commercial sale. Regardless, the ATPase reaction yielded the most linear pH-to-[Product] profile (Figure 9c) and provides an interesting comparison to the HKase reaction (Figure 10c), which also had a near linear profile, albeit with two disparate responses (initial and end). Furthermore, there is an expectation that the rate of pH change for the ATPase reaction should be lower than the other reactions due to the previously documented influence of HPO_4_^−2^ production and pH-dependent H^+^ buffering. This issue will be further explained in “Discussion,” though it is important to raise now to ensure a more complete understanding of the complexity of the results.

The non-linear pH- and [H^+^]-to-[Product] profiles of the CKase (both directions), HKase, and G_6_PDH reactions make pH- and [H^+^]-to-[Product] associations difficult to quantify and interpret. Nevertheless, when fitting data to linear (or close to linear) segments of the pH and [H^+^] responses, some interesting observations were documented. As identified across the figure legends of Figures 7–11 and the data labels with each figure, the pertinent linear segments used are overlaid on the data. The pH-to-[Product] slopes (pH units mmol^-1^ L^-1^ of product formation; -‘ve = H^+^ release and ↓pH) for each reaction are as follows: CKase to ATP = 1.289; CKase to CrP = −1.35; ATPase = −0.9385; HKase = −1.596; G_6_PDHase = −0.8006. Clearly, there is modest consistency among subsets of the pH-to-[Product] profile results that equate to absolute values between 0.8 and 1.7 pH units mmol^-1^ L^-1^ product formation. For all reactions, the most responsive and consistent phase of the pH-to-[Product] profile was the central phase of the pH response. The slope data for the ATPase (Figure 9c), HKase (Figure 10c), and G_6_PDHase reactions (Figure 11c) are pertinent and present the most consistent responses. However, and as expected, the ATPase reaction (Figure 9) yielded a modest rate of pH change, presumably due to the fractional HPO_4_^−2^ H^+^ buffering. Conversely, the highest pH-to-[Product] slope was for the HKase reaction, which is consistent with the highly negative H^+^ coefficient for the reaction (see Table 1 and “Discussion”). See “Discussion” for more details and estimates of the multiple added variables that could account for the variability in this data and for estimates of the influence of HPO_4_^−2^ H^+^ buffering for different pH values during the ATPase reaction.

In summary, the pH and related [H^+^] responses to the H^+^_e_ of product formation for the chemical reactions is not consistent across the wider range of [Product] or pH. However, more research with more sensitive product and pH measurement and with added higher temporal resolution for both variables is needed to ascertain the validity of these observations. The difference between reactions for the pH-specific alteration of enzyme activity across what is a non-physiological pH range must also be considered. These items are addressed in more detail in the “Discussion” section.

### Cuvette reaction data

To confirm the product formation from the pH profile reactions, the product accumulation or decrease in substrate was measured directly from each chemical reaction linked to the increase in NADPH from the G_6_PDHase reaction using enzyme spectrophotometry. As explained in “Methods: (also see Table 5), such data collection could only be acquired from the CKase-to-ATP, -HKase, and -G_6_PDHase reactions due to the involvement of ATP as a substrate in the CKase-to-CrP and -ATPase reactions. The resulting [Product] curves for different dilutions of the reagents for the similar enzyme activity to the pH profile reagent conditions are presented in Figures 12a–c. Note how the production formation from the pH profile data acquisitions for the CKase reaction to ATP are almost identical for both methods based on using the mean value for the different dilutions of the cuvette method reagent versus the single trial test-tube assay method, respectively (∼1.66 vs. 1.95 mmol L^-1^), with similarly near identical results between both methods for the HKase reaction (∼2.16 vs. 2.36 mmol L^-1^). Conversely, the G_6_PDHase reaction revealed higher product formation from the cuvette method (2.53 vs. 1.46 mmol L^-1^) (see “Discussion”).

**FIGURE 12:**
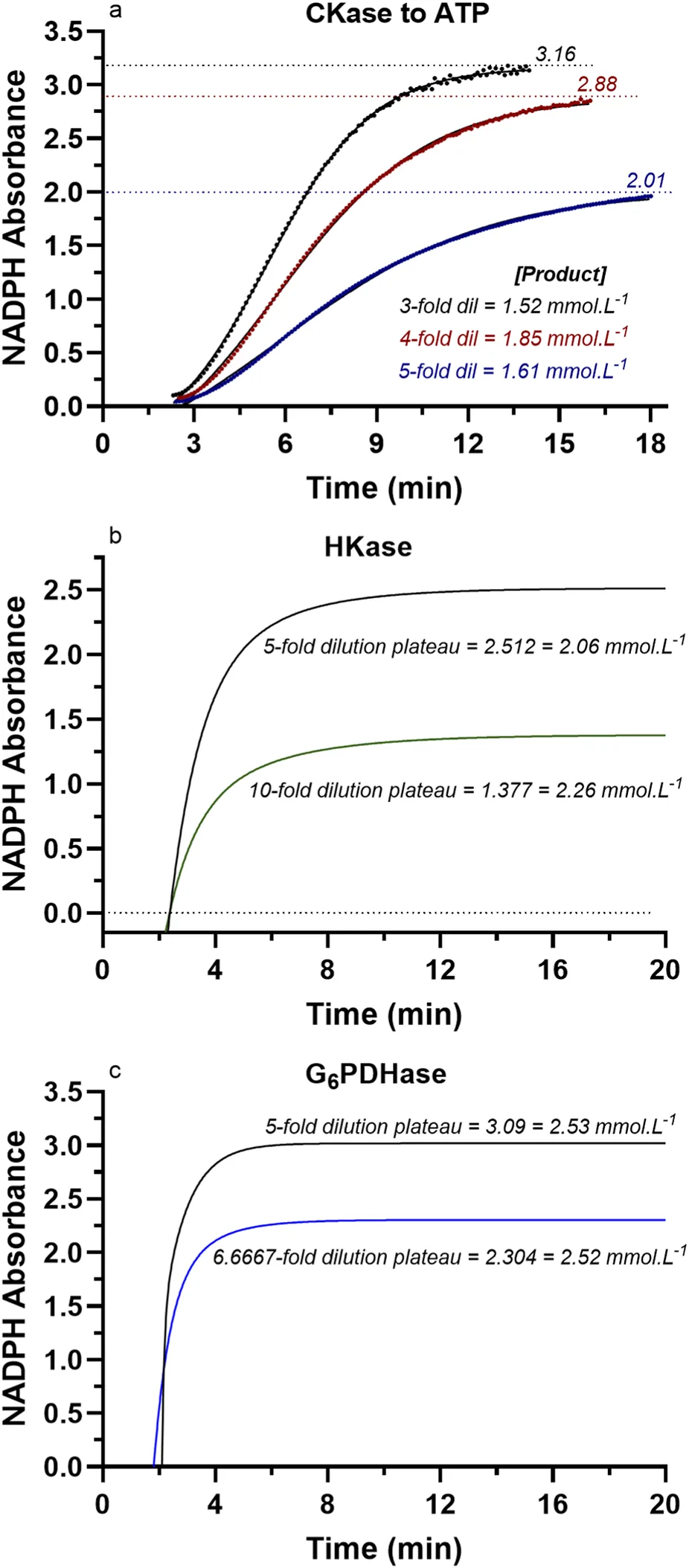
Resulting absorbance increases for the cuvette versions of the chemical reactions for **(a)** CKase reaction to ATP, **(b)** HKase reaction, and **(c)** G_6_PDH reaction (see Table 5). As explained in “Methods,” non-linear curve fitting allowed measurement of the plateau [NADPH] response and thus calculation of the [Product] based on the Beer–Lambert Law and the accompanied dilution factors.

## Discussion

This research investigated the H^+^_e_ and related pH profiles of select non-glycolytic cellular phosphate transfer and oxidation–reduction chemical reactions. Individual reactions were the CKase reaction to ATP and the reverse direction to CrP, and the ATPase, HKase, and G_6_PDHase reactions. Given the clearly stated four purposes of this research, this summary of the results will present the consequent findings linked to such purpose. 1) As with the prior results for the LDH reaction, in either direction to lactate or pyruvate, the chemical reaction H^+^_e_ adhered to the organic and computational chemistry of the reactions. In other words, where the reaction consumed a H^+^, pH increased. Where the reaction released a H^+^, pH decreased (Tables 1, 3; Figure 2). This revealed that the CKase reaction to ATP increased the pH of the aqueous reagent, and each of the CKase reactions to CrP, ATPase, HKase, and G_6_PDHase reactions decreased pH. None of these reactions produced or removed a carboxylic acid intermediate, yet the changes in pH were pronounced. 2) The increased [Product] for each reaction was quantifiable and ranged from 0.84 mmol L^-1^ (ATPase) to 2.36 mmol L^-1^ (HKase), with consistent [Product] resulting from test-tube and direct NADH-linked spectrophotometric methods for all reactions. 3) The pH- and [H^+^]-to-[Product] profiles yielded relatively consistent results across all reactions, though the profiles were not consistent across the range of [Product]. The application of linear regression to segments of the profiles revealed pH-to-[Product] slopes ranging between −1.6 and 1.3 pH units mmol^-1^ L^-1^ product formation, though such results are the beginning of inquiry on this topic and by no means are final or definitive. Interestingly, the pH-to-[Product] slope for the ATPase reaction was moderate at −0.9385 pH units mmol^-1^ L^-1^ product formation, though this was expected due to the stoichiometric production of HPO_4_^−2^, with pK = 6.75. In this regard, the pH-to-[Product] slope for ATPase was prevented from being lower due to the intentional targeted high pH range above the predominant buffering regions of HPO_4_^−2^. As will be further elaborated, the competitive cation influenced fractional H^+^ coefficient for HPO_4_^−2^ → H_2_PO_4_^−1^ at pH 7.0 = 0.359, 7.5 = 0.150, and 8.0 = 0.053 (extrapolated from Robergs (2017)) could be used to adjust the pH response for a given change in [Product], though the process for doing that at this time remains unclear. 4) The influence of HPO_4_^−2^ production from the ATPase reaction to the diminished pH and [H^+^] profiles (to time and to [Product]), in combination to the resulting pH profiles of multiple chemical reactions studied in sequence and simultaneously, revealed pH responses consistent with summative effects of each reaction. Such data reveal the computational potential of the H^+^_e_ of chemical reactions, including buffers and multiple reactions in more complex biological solutions and cells, and may further support the role of altered _a_H_3_O^+^ in the added complexity of Mitchell’s proticity (Mitchell, 1976).

### pH-to-time profiles of individual chemical reactions

Traditional teaching in metabolic biochemistry has viewed H^+^ inclusion in chemical reactions, whether as a substrate or a product, to be indicative of the balanced portrayal of atoms across the reaction. There has never been any interpretation in such textbook presentations that the chemical reaction H^+^_e_ would directly impact the pH of the aqueous environment (Voet and Voet, 2018; Nelson and Cox, 2021). Stewart (1983) assumed this absence of influence as an axiom (an essential, unquestioned assumption) of his physico-chemical computational account for how altered strong ion differences induce changes in the ionization of water, which, in turn, was proposed to cause the changes in pH. However, various studies on skeletal muscle metabolic biochemistry have presented these scenarios differently, where the CKase reaction to ATP production has been interpreted as the cause of the slight alkalinization of skeletal muscle during the initial reactions of repeated muscle contractions, which, in turn, could function to minimize the reductions in pH during cellular metabolism (Roussel et al., 2003; Jones et al., 2008; Walliman et al., 2011; Yquel et al., 2002; Fiedler et al., 2016). Why this view has, until recently (Robergs et al., 2004; Robergs, 2017; Robergs, 2019; Robergs, 2021; Robergs, 2017), never transferred to how the H^+^ consumption of lactate production must therefore also metabolically buffer against large decreases in pH remains a mystery. Such contradictory evidence to both the metabolic acid conventional interpretation of the cause of metabolic acidosis and to the Stewart approach has stimulated some added research or scholarship (Aiken, 2013; Sirker et al., 2012), though with minimal impact in establishing sufficient evidence to allow a dominance of one theory or paradigm (Kuhn, 2012) over another for the metabolic causes of metabolic acidosis in animal systems. What has been missing in prior research into chemical reactions, in both animal and plant-based systems, is the direct cause–effect evidence for how chemical reaction H^+^_e_ influences the pH of the aqueous environment. Our current research and results have addressed this.

### CKase reactions

The CKase reaction is a classic example of a near-equilibrium reaction *in vivo*, where if [Substrates] are increased and/or [Products] are decreased) (*in vivo* or *in vitro*), the direction of the chemical reaction can change. The estimates of the free energy exchange of the CKase reaction *in vivo* (ΔG) are presented in Table 1. Such chemical reaction biophysics and energetics were exploited in this study by the controlled preparation of reagents for the CKase reaction for ATP formation (no [ATP] or [Cr] present in the prepared reagent) versus CrP formation (no [CrP] or [ADP] present in the prepared reagent) (Table 1).

The CKase enzyme has wide isozyme variation, with varied enzyme structures and slightly different kinetics across the different isozymes. CKase located in the cytosol is a dimer consisting of two subunits that are denoted as either muscle (M) or brain (B) monomers. Consequently, cytosolic CKase can exist as MM-CK, BM-CK, or BB-CK isozymes (Schlattner et al., 1998; Shen et al., 2001). There is also a structurally different mitochondrial CKase (Mt-CK), with a more complex structure consisting of eight independent monomers arranged as four banana-shaped dimers (Schlattner et al., 1998; Shen et al., 2001). There has been difficulty in forming crystal structures of CKase in order to ascertain the mechanisms of the reaction. Despite this, Li et al. (2016) provide an excellent detailed account of the reaction mechanism for Mt-CK. Consequently, our results confirm the interpretations of others of the importance of chemical reactions that involve H^+^_e_ in the acid-base balance of their aqueous environment and, in extension, in cellular and more holistic physiological systems.

The involvement of CK reactions (either direction) in the regulation and response of metabolism to increased energy demand and alterations to pH is even more important when the broader roles of the CKase reaction in mitochondrial to cytosolic free energy and phosphate transfer is understood (creatine kinase shuttle) (Figure 3). Such involvement reveals that the CKase reaction can no longer be viewed as a reaction confined to periods of intense ATP demand. The reversibility of the CKase reaction and its oppositional H^+^e during such free energy mitochondrial to cytosolic energy transfer allows for minimal effects to net pH changes while further supporting both ADP and H^+^ transfer to the mitochondria. Conversely, the dominance of the CKase reaction to ATP formation in times of high ATP demand occurs with a net H^+^ consumption (+’ve H^+^_e_), suggesting a meaningful role in cellular H^+^ metabolic buffering during conditions of increased glycolytic ATP turnover and net H^+^ release (Robergs, 2017; 2019; 2021).

### ATPase reaction

As presented in Table 1 and Figure 1, the ATPase reaction involves the release of a phosphate group from ATP that forms ADP and a free HPi (HPO_4_^−2^). As the cleavage of the HPi required the addition of a hydroxyl group (-OH), this comes from the splitting (hydrolysis) of water, thereby releasing a H^+^ to solution. This is the source of the H^+^ that causes the decrease in pH resulting from this reaction. Being a life-dependent enzyme crucial across a wide variety of physiological processes, the Na^+^,K^+^-ATPase enzyme is highly researched. Due to the cellular and physiological system specificity of the enzyme, there are many genetically expressed isozymes and additional protein structures that afford such specialization. Nevertheless, it is best to emphasize a few details of this variability to further clarify the explanation of the results of this research.

The ATPase enzymes consist of one alpha and one beta isoform, along with additional regulatory and function specific protein residues (Clausen et al., 2017; Neupane et al., 2019). There are four different alpha isoforms and three different beta isoforms. For the Na^+^-K^+^-ATPase—the type of ATPase used in this research (Table 1) —the catalytic mechanism involves a water molecule that provides the charge negativity to facilitate the removal of the terminal (gamma) phosphate group of ATP while then providing a hydroxyl (-OH) group from the water molecule resulting from the hydrolysis to complete the phosphate group structure, leaving the remaining H^+^ to be released (Aperia et al., 2016; Ogrizek et al., 2022). Such enzyme activity is dependent on the binding of Na^+^ and K^+^ to the alpha subunit, and there are multiple phases of the cycle of ATP hydrolysis linked to the multiple phases of the operation of the connected Na^+^-K^+^ pump (Aperia et al., 2016; Ogrizek et al., 2022).

Figure 2c documents wide variability in the pH-to-time profiles of the ATPase reaction. Prior explanations in “Methods” and “Results”, combined with the content above, reveals that such variability was intentionally acquired to show the importance of the roles of Na^+^ and K^+^ as activators of the Na^+^,K^+^-ATPase and where the pH_start_ was important due to one of the products of the reaction being the H^+^ buffer HPO_4_^−2^ (pK = 6.75). Thus, the [HPO_4_^−2^] is stoichiometric to the [ADP] shown in Figure 9b. Given that we know the pH values across the [Product] range, we can calculate the H^+^ buffering by HPO_4_^−2^ across this response. We obtained the dissociation constant and the data sets for H^+^ buffering by HPO_4_^−2^ from Robergs (2017). As the published data was constrained to a pH range from 6.0 to 7.0, we broadened the pH range to 9.0 to suit the current research. The results revealed fractional values for H_2_PO_3_ for pH = 6.0 to 9.0 to be 0.8483 to 0.055, respectively. For the pH range covered in Figures 9a and c (pH = 8.0–9.0), the fractional values ranged from 0.0529 to 0.0055, respectively. For this range of pH-dependent fractional H^+^ buffering by HPO_4_^−2^, further adjusted for [Product] (Figure 9b) (the peak [Product] accumulation (0.84 mmol·L^-1^) at the corresponding pH value (8.23)) (see Table 3, ATP7), this amounts to a total H_2_PO_4_^−1^ H^+^ fraction (F_H_ = 0.0319) and buffering of 26.8 μmol·L^-1^ (see Equation 2). Due to the intentionally targeted high pH of this reaction condition, this is a relatively low H^+^ buffering value for the given production of HPO_4_^−2^.

$$H^{+}\;Buffering=peak\;\left[Product\right]\times F_{H}.$$ (2)

When applied to a lower pH range, as presented in Figure 2c ATP5 (Table 3, ATP5), and assuming the same final [Product], the final pH was 7.28, with a H_2_PO_4_^−1^ F_H_ = 0.2270 and a H^+^ buffering of 190.7 μmol·L^-1^. Both resulting values for H^+^ buffering appear low, but these values need to be adjusted for the influence of H^+^ to pH. When applying Equation 1 to such H^+^ buffering values, current estimates of the resulting pH changes that could result without the buffering for the stated measured final pH value amount to a pH reduction from 8.23 to 5.81 for the data of ATP7 and a pH change from 7.28 to 4.0 for ATP5. As identified by Robergs et al. (2026), such estimated values based on Equation 1 are too large to be reasonable pH consequences of the H^+^_e_ of chemical reactions for these [Product] values. As such, further research into the magnitude of the pH change to the magnitude of the H^+^_e_ of chemical reactions is needed to ascertain a valid computation of their direct impact on pH. This is even more important for the narrow cell physiological range of pH (6.0–7.0). We are already at work to contribute to this needed area of research (see the subsection on *Computational Relevance*).

### The HKase reaction

The hexokinase reaction catalyzes the ATP-dependent phosphorylation of glucose to glucose-6-phosphate and ADP and, in the process, also releases a H^+^ into solution (Table 1). Unlike the ATPase reaction, there is no hydrolysis accompanying this reaction. Rather, the terminal (gamma; λ) phosphate group of ATP is not released to solution but transferred to the carbon-6 (C_6_) of glucose, which requires the splitting of a hydroxyl group bound to the C_6_ of glucose. The phosphate from ATP connects to the remaining oxygen atom, releasing a H^+^ to solution (Rose, 1980; Voet and Voet, 2018; Nelson and Cox, 2021; Kuser et al., 2000; Mai and Meere, 2021).

Due to its diverse importance in cellular metabolism across multiple tissues and a component of blood glucose regulation by the liver, hexokinase is a well-researched enzyme. There are four different isozymes of hexokinase, all with numeric suffixes; *I*, *II*, *III*, and *IV*. Hexokinase-*I* is present in all tissues; hexokinase-*II* is mainly expressed in skeletal muscle and adipose tissue; hexokinase-*III* is broadly expressed in low levels across all tissues, with relatively larger expression, though still low, in the lungs, kidneys, and liver; hexokinase-*IV* (known generically as “glucokinase”) is expressed in the liver and pancreas β-cells (Kuser et al., 2000; Mai and Meere, 2021). The hexokinase used in this research was from yeast, with a predominant expression of hexokinase-*I* and -*II*.

The computational chemistry of the hexokinase reaction reveals net H^+^ release (-‘ve H^+^_e_), where Robergs (2017) documented a H^+^_e_ for pH = 6 and 7.0 to be −0.258 and −0.850, respectively. However, a minor calculation error overlooked the unitary H^+^_e_ of the reaction, and the corrected values for these pH conditions are −1.258 and −1.850, respectively (Table 1). Thus, the hexokinase reaction in the direction of G_6_P production is highly H^+^-releasing. Due to the low [G_6_P] of the cytoplasm of most extrahepatic cells (it is removed to form glycogen during high carbohydrate conditions and is removed by glycolysis during high energy-demand conditions), the reaction is highly exergonic in the direction of G_6_P (Table 1) (Meurer et al., 2016). The absence of G_6_P inhibition of hexokinase-*IV* enables the enzyme to remain active in hepatocytes as G_6_P increases during times of diminished carbohydrate availability and raised gluconeogenesis. Thus, during these conditions, the bioenergetics of the reaction change dramatically and favor the reverse direction of the reaction to now be the exergonic one. Added kinetic differences between hexokinase-*IV* and the other isozymes further support this unique role in the liver.

From the perspective of metabolic acidosis during conditions of increased energy demand, the hexokinase-*I*, -*II* and -*III* reactions contribute to the net H^+^ release of glycolysis. The reaction (Figure 12) produced a relatively large [Product], and the range of pH change of close to 2 pH units was far higher than all other reactions other than the CKase reaction to ATP (Figure 9). Interestingly for conditions where glycolysis is fueled by glycogenolysis, this H^+^ release does not occur for the related G_6_P flux through glycolysis. Consequently, the opposing H^+^_e_ of ATP hydrolysis and lactate production would be highly influential on the extent of cellular acidosis.

### G_6_PDHase reaction

The G_6_PDHase reaction, as with the lactate dehydrogenase (LDHase) reaction, is an oxidation–reduction reaction. By definition, such reactions have a substrate (reductant) that releases electrons (oxidation) and another substrate (oxidant) that receives (gains) the electrons (reduction) to form a different product. For the LDHase reaction to lactate, the reductant is NADH and the oxidant is pyruvate. For the G_6_PDH reaction, the reductant is G_6_P and the oxidant is NADP^+^. Though both the LDHase and G_6_PDHase reactions are exergonic to a favored direction, the directions are different in the context of the oxidation–reduction. For G_6_PDHase, the exergonic direction is that leading to 6-phosphogluconolactone and NADPH. Furthermore, *in vivo*, the G_6_PDHase reaction is made highly exergonic in the aforementioned direction due to rapid conversion of 6-phosphogluconolactone to 6-phosphogluconate, thereby essential making this a unidirectional (non-reversible) reaction (Rossi et al., 2003).

The H^+^_e_ occurs due to the removal of two electrons and 2 H^+^ from C_1_ of G_6_P in the process of oxidation. The two electrons and 1 H^+^ are involved in the reduction of NADP^+^ to NADPH, leaving the remaining H^+^ to be released and having its effect on the pH of the solution (Nelson and Cox, 2021; Sun et al., 2024).

Figure 11 reveals slightly different pH-time and [Product] profiles to that from the HKase reaction, though of course, the features of the pH-time and the [Product]-time profiles are highly influenced by the selected [Substrate] and enzyme activity conditions. Regardless, as for the prior reactions, similar pH or [H^+^] to [Product] profiles across all the reactions would be expected given that they are expressed relative to [Product]. The pH and [H^+^] to [Product] profiles will be discussed in more detail below. It is thus noteworthy to stress the consistency of the pH responses to the organic chemistry of each reaction. This feature becomes even more interesting in the next section, where the results from sequenced and combined reactions further behave as predicted from the summative features of the multiple reactions.

### pH-to-time profiles of sequenced and simultaneous chemical reactions

The study of the H^+^_e_ of multiple reactions is a logical progression from the original focus of research on the LDHase reaction (Robergs et al., 2026). The results document the predictable and summative feature of the H^+^_e_ of chemical reactions that have similar or opposing directional H^+^_e_. Such responses are documented in Figures 4–6. Figure 4 reveals the more rapid decrease in pH once the ATPase reaction was added to the CKase reaction in the direction of CrP production. As previously explained, the pH commenced at 9.23 (Table 3) due to the need to document the pH profile without the H^+^ buffering by HPO_4_^−2^ as a product of the ATPase reaction.

Such predictable behavior of pH to the H^+^_e_ of multiple chemical reactions is further documented in Figure 5 for added scenarios for the operation of the CKase reaction to ATP sequenced to the ATPase reaction across longer time periods. Addition of the ATPase reaction to the pH plateaued response of CKase to ATP revealed that the pH response to the ATPase reaction was still blunted (Figures 5a,b). This is to be expected as the ATPase reaction, producing ADP, provides added substrate to further support continued activity of the CKase reaction to ATP.

Figures 5c,d compares the pH response to the CKase reaction to ATP simultaneous with the ATPase reaction. Compared to the ATPase reaction in isolation, the pH response was highly blunted. Figure 5e emphasizes the importance of the reagent pH to the pH responses, where the sequencing of the CKase reaction to CrP failed to show the expected magnitude of the pH decrease once the ATPase reaction was added for reagent pH conditions < 8.2. Such summative responses of pH to combined reactions were further explored in the sequencing of the CKase reaction to ATP with the HKase and G_6_PDHase reactions (Figure 6). The staircase response to pH was profound and collectively larger due to the increased pH of the prior CKase reaction to ATP, compared to the combined condition of all three reactions.

Such sequenced and simultaneous chemical reaction pH profile results may also reveal the more complex roles of altered and intracellular gradients in _a_H_3_O^+^ in facilitating near immediate shuttling of H^+^ from one reaction to another. Such a possible function stresses the need more deeply consider the thermodynamics and biophysics of the ionization properties of water and its role in facilitating such proticity, as was originally proposed by Mitchell (1976). Indeed, the smooth and near immediate nature of the pH responses to these combined reactions (Figures 2 and 6), though complicated by the relatively slow temporal response output of the pH electrode, may reveal further evidence for the function of such proticity in the chemical reactions and pH regulation of cellular cytosolic metabolic systems.

We have yet to provide a computational account of these responses. We aim to do that in future research. For now, our intent is to document these responses and provide some direction to the computational strategies that might provide an accurate account of such pH behavior. We expand on this issue in the section concerning the pH- and [H^+^]-to-[Product] profiles.

### Product accumulation responses

To better interpret the pH-time profiles of the five chemical reactions, it was necessary to establish that each reaction occurred by quantifying product formation and, as such, that the pH changes were linked to product formation. Similar methods and results have been shown for the LDHase reaction to either lactate or pyruvate (Robergs et al., 2026). Such product formation was assessed from two different methods. The first was from traditional enzyme spectrophotometry, where samples of the reagent acquired during the select pH-time profile trials of each reaction were assayed for total product. These results were further verified from the kinetics of the direct production or removal of NADPH via enzyme-linked spectrophotometry. This direct procedure involved the pipetting of 1 mL of the reagent into a crystal cuvette, where once again the reaction was initiated by the addition of the enzyme, along with other enzymes (either or both of HKase and G_6_PDHase) needed to link the reaction products to a change in the [NADPH] within the cuvette (see Table 1).

The product formation results from the pH–time profile trials of the five reactions are presented in Figures 7b–11b. All have slightly different kinetic profiles, as expected from the different enzymes of the reactions, the enzyme activity units used, and slightly different [Substrates] and pH_start_ values. The added relevance of these results is revealed in the next section.

### pH- and [H^+^]-to-[Product] profiles

Robergs et al. (2026) reported pH: [Product] slopes (pH mmol^-1^ L^-1^) for the D-LDHase reaction of 9.215 for D-lactate production and −9.074 for pyruvate production. Such slopes are vastly different for the results of this research, where slopes ranged from 1.3 to −1.6. At this time, it is difficult to account for such a large difference, though as detailed by Robergs et al. (2026), added factors must be explored and quantified such as the H^+^ buffering by the substrates and products of the reaction, added buffering by the enzyme, and as yet uncertain influences of the direct chemical reaction of H^+^e to the ionization of water at different ionic strength conditions. Such factors require further research.

What is noticeably different in this research from Robergs et al. (2026) is the larger [Product] formation from each of the five reactions studied. The data for [Product] are subsequently more detailed in documenting clear linear and non-linear functions, presumably made more definitive through the added accuracy of the assays for higher [Product]. More research of these and other chemical reactions is needed to permit better understanding of the expectations for and influences on such pH: [product] slope responses.

### Computational Relevance of chemical reaction H^+^_e_

Results for the pH- and [H^+^]-to-[Product] profiles revealed varied responses, where some evidence existed to support a linear response of either of pH and [H^+^] across segments of the total [Product] profiles. However, as with the LDH reaction (Robergs et al., 2026), there was unexplained inconsistency in this response between the different chemical reactions. As for the prior research of the LDH reaction (Robergs et al., 2026), peak [Product] accumulation remained small, where for CKase-to-ATP, CKase-to-CrP, ATPase-to-ADP, HKase G_6_P and G_6_PDH-to-6PGl, the [main product] data were 1.95, 0.64, 0.84, 2.26, and 1.46 mmol·L^-1^, respectively (Table 3).

As explained in “Results” for each chemical reaction, the data for the pH- and [H^+^]-to-[Product] profiles are very important to further understand the influence of the H^+^_e_ of chemical reactions on the acid–base balance of the aqueous environment of the reaction. The sequenced and simultaneous CKase (to either direction of ATP or CrP) and ATPase reactions (Figures 4, 5) documented predicted directional alterations to the changes in pH based on the organic and computational chemistry of the reactions. Such responses were further reinforced by the results of Figure 6, where the CKase reaction to ATP was progressively sequenced to the addition of the HKase reaction and then the G_6_PDHase reaction. The [Product] was not measured in these trials, though clearly, based on the prior [Product] results for individual chemical reactions, this needs to be done to shed further understanding on the underlying chemistry of direct H^+^ involvement to the pH of an aqueous system. For now, the results are highly encouraging for the potential of a computational account of the pH changes when there is either an addition or removal of H^+^ from solution.

## Conclusions, limitations, and recommendations

This research expanded on the prior research of Robergs et al. (2026), who studied the influences of the H^+^_e_ of the LDHase reaction to lactate and the reverse direction or pyruvate. The LDHase reaction to lactate increased pH and the reaction to pyruvate decreased pH. The findings of this research revealed the direction and magnitude of changes to pH for the CKase reaction (to ATP and to CrP), ATPase, HKase, and G_6_PDHase reactions. As for the prior research of the LDHase reaction, all pH responses for the chemical reactions of this research adhered to the organic and computational chemistry of the chemical reactions, where the role of the H^+^ as a substrate caused an increase in pH (CKase to ATP), and its role as a product caused a decrease in pH (CKase to CrP, ATPase, HKase, and G_6_PDH reactions). The changes to pH relative to [Product] were similar across all reactions, especially considering the difficulty in accurately measuring small increases in [Product] across short time intervals. Future research needs to correct these pH responses for pH-dependent H^+^ association and dissociation differences between substrates and products using ionic strength-adjusted dissociation constants.

There were two additional concerns for this research. The first was the temporal insensitivity of the pH meter to rapid changes in pH. Such rapid changes occurred during the initial minutes of some chemical reactions, rendering the accurate measurement of each of pH and [Product] difficult. This issue was addressed by lowering the enzyme activity and [Substrate] of the reagent, which, in turn, lowered the rate of product formation. However, as previously explained, such corrections lowered [Product] results. Such responses may be resolved by using a more sensitive method of product measurement, such as product-specific liquid chromatography tandem mass spectrometry. The second was the likelihood for extreme changes in reagent pH to decrease enzyme catalytic activity, product formation, and resultant changes in pH. We minimized this issue by not including the final segment of pH data in our analyses of the pH–[Product] profiles.

The results are compelling for the acid–base chemistry and physiology of animal systems. Such combined research of both pH and [Product] for isolated chemical reactions has been absent in the epistemology of both disciplines for more than 100 years. The results document the need for more research on this topic across the same and additional chemical reactions, and using this information to learn more about cellular metabolic contributors to cellular and systemic acid-base perturbations. In this context, in addition to pH measurement, there is need to quantify the diminishing capacity of a buffer system, which would essentially be a measure of the quantity of H^+^ buffered. The pH–[Product] data are also interesting in their potential for devising a new computational approach for understanding and predicting the pH changes for different metabolic conditions. Indeed, knowing the full extent of gross H^+^ release and consumption during cellular metabolism allows a more correct estimate of needed H^+^ buffering for any condition of metabolic demand in addition to further documenting the potential of Mitchell’s proticity theory to further our understanding of not just biological acid–base chemistry but also the broader involvement of aH_3_O^+^ in the cellular regulation of energy transfer and work. We encourage other researchers to also contribute to this important extension of this research.

The results of this research expand on prior evidence (Robergs et al., 2026) for understanding individual chemical reactions. Thus, there is scope to regulate chemical reactions in cellular or tissue-based experimental models and quantify either or both of the pH responses and altered H^+^ buffer capacity. Given that science concerns discovery as a means to advance knowledge, the results of this research are encouraging of such future scientific exploration of the biochemical contributions, further encompassing multiple added physiological systems, of one of the life-dependent regulatory systems; the pH regulation of biological solutions.

## Data Availability

The raw data supporting the conclusions of this article will be made available by the authors without undue reservation.

## References

[B1] Aiken C. G. A. (2013). History of medical understanding and misunderstanding of acid base balance. J. Clin. Diagn Res. 7 (9), 2038–2041. 10.7860/JCDR/2013/5230.3400 24179938 PMC3809677

[B2] Aperia A. Akkuratov E. E. Fontana J. M. Brismar H. (2016). Na^+^-K^+^-ATPase, a new class of plasma membrane receptors. Am. J. Physiol. Cell Physiol. 310, C491–C495. 10.1152/ajpcell.00359.2015 26791490

[B3] Clausen M. V. Hilbers F. Poulsen H. (2017). The structure and function of the Na,K-ATPase isoforms in health and disease. Front. Physiol. 8, 371. 10.3389/fphys.2017.00371 28634454 PMC5459889

[B4] De Ruyck J. Fameree M. Wouters J. Perpete E. A. Preat J. Jacquemin D. (2007). Towards the understanding of the absorption spectra of NAD(P)H/NAD(P)^+^ as a common indicator of dehydrogenase enzymatic activity. Chem. Phys. Lett. 450 (1-3), 119–122. 10.1016/j.cplett.2007.10.092

[B5] Fiedler G. B. Schmid A. I. Goluch S. Schewzow K. Laistler E. Niess F. (2016). Skeletal muscle ATP synthesis and cellular proton handling measured by localized 31P-MRS during exercise and recovery. Sci. Rep. 6, 32037. 10.1038/srep32037 27562396 PMC4999956

[B7] Goldberg R. N. (1975). Thermodynamics of hexokinase-catalyzed reactions. Biophys. Chem. 3 (3), 192–205. 10.1016/0301-4622(75)80011-1 240453

[B8] Golding E. M. Teague W. E. Dobson G. P. (1995). Adjustment of K’ to varying pH and pMg for the creatine kinase, adenylate kinase and ATP hydrolysis equilibria permitting quantitative bioenergetic assessment. J. Exp. Biol. 198 (8), 1775–1782. 10.1242/jeb.198.8.1775 7636446

[B9] Hill A. V. Lupton H. (1923). Muscular exercise, lactic acid, and the supply and utilization of oxygen. QJM Ann. Int. J. Med. 16 (62), 135–171. 10.1093/qjmed/os-16.62.135

[B10] Jones A. M. Wilkerson D. P. DiMenna F. Fulford J. Poole D. C. (2008). Muscle metabolic responses to exercise above and below the critical power| assessed using ^31^P-MRS. Am. J. Physiol. Regul. Integr. Comp. Physiol. 294, R585–R593. 10.1152/ajpregul.00731.2007 18056980

[B11] Katz A. Sahlin K. (1988). Regulation of lactic acid production during exercise. J. Appl. Physiol. 65 (2), 509–518. 10.1152/jappl.1988.65.2.509 3049511

[B12] Kragh H. Sorenson S. P. L. (2025). The pH concept and its early history. Found. Chem. 27, 237–261. 10.1007/s10698-025-09532-6

[B13] Kreisberg R. A. (1980). Lactate homeostasis and lactic acidosis. Ann. Int. Med. 92 (21), 227–237. 10.7326/0003-4819-92-2-227 6766289

[B14] Kuhn T. (2012). The Structure of Scientific Revolutions, 4th Edn, The University of Chicago Press, Chicago, IL.

[B15] Kuser P. R. Krauchenco S. Antunes A. O. C. Polikarpov I. (2000). The high resolution crystal structure of yeast hexokinase PII with the correct primary sequence provides new insights into its mechanism of action. J. Biol. Chem. 275 (27), 20814–20821. 10.1074/jbc.M910412199 10749890

[B16] Kushmerick M. J. (1997). Multiple equilibria of cations with metabolites in muscle bioenergetics. Am. J. Physiol. 272 (51), C1739–C1747. 10.1152/ajpcell.1997.272.5.C1739 9176167

[B17] Le X. Wu F. Qi F. Beard A. (2011). A database of thermodynamic properties of the reactions of glycolysis, the tricarboxylic acid cycle, and the pentose phosphate pathway. Database 2011, bar005. 10.1093/database/bar005 21482578 PMC3077827

[B18] Lehninger A. L. (1960). Energy transformation in the cell. Sci. Am. 202 (5), 102–117. 10.1038/scientificamerican0560-102 14415395

[B19] Lehninger A. L. (1961). How cells transform energy. Sci. Am. 205 (3), 62–73. 10.1038/scientificamerican0961-62 13760489

[B20] Lesney M. S. (2003). A basic history of acid – from Aristotle to Arnold. Today’s Chem. at Work March, 47–48.

[B21] Li Q. Fan S. Li X. Jin Y. He W. Zhou J. (2016). Insights into the phosphoryl transfer mechanism of human uqiquitous mitochondrial creatine kinase. Sci. Rep. 6, 38088. 10.1038/srep38088 27909311 PMC5133464

[B22] LoPresti P. Cohn M. (1989). Direct determination of creatine kinase equilibrium constants with creatine or cyclocreatine as substrate. Biochem. Biophysica Acta 998, 317–320. 10.1016/0167-4838(89)90291-4 2804134

[B23] Lowry O. H. Passeneau J. V. (1972). A Flexible System of Enzymatic Analysis. Orlando, FL: Academic Press.

[B24] Mai V. Q. Meere M. (2021). Modelling the phosphorylation of glucose by human hexokinase I. Mathematics 9, 2315. 10.3390/math9182315

[B25] Meurer F. Bobrownik M. Sadowski G. Held C. (2016). Standard Gibbs energy of metabolic reactions: I. Hexokinase reaction. Biochemistry 55 (40), 5665–5674. 10.1021/acs.biochem.6b00471 27653185

[B26] Mitchell P. (1976). Vectoral chemistry and the molecular mechanics of chemiosmotic coupling: power transmission by proticity. Biochem. Soc. Trans. 4 (3), 399–430. 10.1042/bst0040399 137147

[B27] Nelson D. L. Cox M. M. (2021). Lehninger Principles of Biochemistry. 8th Edn. International, New York, NY: Macmillan.

[B28] Neupane P. Bhuju S. Thapa N. Bhattarai H. K. (2019). ATP synthase: structure, function and inhibition. Biomol. Concepts 10, 1–20. 10.1515/bmc.2019.0001 30888962

[B30] Ogrizek M. Janezic M. Valjavec K. Perdih A. (2022). Catalytic mechanisms of ATP hydrolysis in the ATPase domain of human DNA Topoisomerase IIa. J. Chem. Info Model 62, 3896–3909. 10.1021/acs.jcim.2c00303 35948041 PMC9400105

[B31] Robergs R. A. (2017). Competitive cation binding computations of proton balance for reactions of the phosphagen and glycolytic energy systems within skeletal muscle. PLoS One 12 (12), e0189822. 10.1371/journal.pone.0189822 29267370 PMC5739460

[B32] Robergs R. A. (2019). Invited review: quantifying proton exchange from chemical reactions - implications for the biochemistry of metabolic acidosis. Comp. Biochem. Physiol Part A Mol Integrat Physiol 235, 29–45. 10.1016/j.cbpa.2019.04.024 31071454

[B33] Robergs R. A. (2021). Quantifying H^+^ exchange from muscle cytosolic energy catabolism using metabolite flux and H^+^ coefficients from multiple competitive cation binding. Physiol. Rep. 9 (7), e14728. 10.14814/phy2.14728 33904663 PMC8077081

[B34] Robergs R. A. Ghiasvand F. Parker D. (2004). Biochemistry of exercise-induced metabolic acidosis. Am. J. Physiol. Regul. Integr. Comp. Physiol. 287, R502–R516. 10.1152/ajpregu.00114.2004 15308499

[B35] Robergs R. A. Nalos M. Kelbich P. Jizerova A. Mervartova I. Vostry M. (2026). D- and L-Lactate dehydrogenase reactions increase pH to lactate production and decrease pH to pyruvate production. Front. Mol. Biosci. 13, 1715499. 10.3389/fmolb.2026.1715499 42022288 PMC13095559

[B37] Roosterman D. Cottrell D. S. (2025). Mechanisms of glycolysis and fermentation: A non-equilibrium thermodynamics perspective. Qeios. 10.32388/Y9K21X.2

[B39] Rose I. A. (1980). Mechanism of phosphoryl transfer by hexokinase. Biochem. Biophys. Res. Comm. 94 (2), 573–578. 10.1016/666-291X(80)91270-X 6994724

[B40] Rossi F. G. Ribeiro M. Z. Converti A. Vitolo M. Pessoa A. Jr (2003). Kinetic and thermodynamic aspects of glycose-6-phosphate dehydrogenase activity and synthesis. Enzyme Microbiol. Tech. 32, 107–113. 10.1016/s0141-0229(02)00242-9

[B41] Roussel M. Mattei J. P. Le Fur Y. Ghattas B. Cozzone P. J. Bendahan D. (2003). Metabolic determinants of the onset of acidosis in exercising human muscle: a 31P-MRS study. J. Appl. Physiol. 94, 1145–1152. 10.1152/japplphysiol.01024.2000 12433845

[B42] Sakano K. (1998). Revision of biochemical pH-Stat: involvement of alternative pathway metabolisms. Plant Cell Physiol. 39 (5), 467–473. 10.1093/oxfordjournals.pcp.a029393

[B43] Schlattner U. Forstner M. Eder M. Stachowiak O. Fritz-Wolf K. Wallimann T. (1998). Functional aspects of the x-ray structure of mitochondrial creatine kinase: a molecular physiology approach. Mol. Cell Biochem. 184, 125–140. 10.1023/A:10068513301913 9746317

[B57] Seheult J. Fitzpatrick G. Boran G. (2017). Lactic acidosis: an update. Clin. Chem. Lab Med. 55 (3), 322–333. 10.1515/cclm-2016-0438 27522622

[B45] Shen Y. Tang L. Zhou H. Lin Z. (2001). Structure of human muscle creatine kinase. Biol. Crystallogr. D57, 11196–11200. 10.1107/S0907444901007703 11517911

[B46] Sirker A. A. Rhodes A. Grounds R. M. Bennett E. D. (2012). Acid-base physiology: the ‘traditional’ and the ‘modern’ approaches. Anaesthesia 57, 348–356. 10.1046/j.0003-2409.2001.02447.x 11939993

[B47] Stewart P. A. (1983). Modern quantitative acid-base chemistry. Can. J. Physiol. Pharmacol. 61, 1444–1461. 10.1139/y83-207 6423247

[B48] Sun L. Sun B. Chen K. (2024). Kinetic properties of glucose 6-phosphate dehydrogenase and inhibition effects of several metal ions on enzymatic activity *in vitro* and cells. Sci. Rep. 14, 5806. 10.1038/s41598-024-5650306 38461203 PMC10924972

[B50] Torrens S. Robergs R. A. Curry S. C. Nalos M. (2023). The computational acid-base chemistry of hepatic ketoacidosis. Metabolites 13 (7), 803. 10.3390/metabo13070803 37512510 PMC10383603

[B51] Trinh M. D. L. Masuda S. (2022). Chloroplast pH homeostasis for the regulation of photosynthesis. Front. Plant Sci. 13, 919896. 10.3389/fpls.2022.919896 35693183 PMC9174948

[B52] Vinnakota K. Kemp M. L. Kushmerick M. J. (2006). Dynamics of muscle glycogenolysis modelled with pH time course computation and pH-dependent reaction equilibria and enzyme kinetics. Biophys. J. 91 (4), 1264–1287. 10.1529/biophysj.105.073296 16617075 PMC1518652

[B53] Voet D. Voet J. G. (2018). Biochemistry. 4th Edn. Hoboken, NJ: John Wiley & Sons, Inc.

[B54] Walliman T. Tokarska-Schlattner M. Schlattner U. (2011). The creatine kinase system and pleiotropic effects of creatine. Amino Acids 40, 1271–1296. 10.1007/s00726-011-0877-3 21448658 PMC3080659

[B55] Wiseman R. W. Brown C. M. Beck T. W. Brault J. Reinoso T. R. Shi Y. (2023). Creatine kinase equilibration and ΔG_ATP_ over an extended range of physiological conditions: implications for cellular energetics, signaling, and muscle performance. Int. J. Mol. Sci. 24, 13244. 10.3390/ijms241713244 37686064 PMC10487889

[B56] Yquel R. R. Arsac L. M. Thiaudiere E. Canioni P. Manier G. (2002). Effect of creatine supplementation on phosphocreatine resynthesis, inorganic phosphate accumulation and pH during intermittent maximal exercise. J. Sport Sci. 20 (5), 427–437. 10.1080/026404102317366681 12043832

